# Epigenetic silencing of miR-145-5p contributes to brain metastasis

**DOI:** 10.18632/oncotarget.5930

**Published:** 2015-09-30

**Authors:** Sara Donzelli, Federica Mori, Teresa Bellissimo, Andrea Sacconi, Beatrice Casini, Tania Frixa, Giuseppe Roscilli, Luigi Aurisicchio, Francesco Facciolo, Alfredo Pompili, Maria Antonia Carosi, Edoardo Pescarmona, Oreste Segatto, Greg Pond, Paola Muti, Stefano Telera, Sabrina Strano, Yosef Yarden, Giovanni Blandino

**Affiliations:** ^1^ Translational Oncogenomics Unit, Italian National Cancer Institute ‘Regina Elena’, Rome, Italy; ^2^ Molecular Chemoprevention Unit, Italian National Cancer Institute ‘Regina Elena’, Rome, Italy; ^3^ Department of Pathology, Italian National Cancer Institute ‘Regina Elena’, Rome, Italy; ^4^ Takis s.r.l., Roma, Italy; ^5^ Unit of Thoracic Surgery, Italian National Cancer Institute ‘Regina Elena’, Rome, Italy; ^6^ Department of Neurosurgery, Italian National Cancer Institute ‘Regina Elena’, Rome, Italy; ^7^ Laboratory of Cell Signaling, Italian National Cancer Institute ‘Regina Elena’, Rome, Italy; ^8^ Department of Oncology, Faculty of Health Science, McMaster University, Hamilton, Canada; ^9^ Weizmann Institute of Science, Department of Biological Regulation, Rehovot, Israel

**Keywords:** brain metastases, lung cancer, mir-145-5p, epigenetic modifications, migration

## Abstract

Brain metastasis is a major cause of morbidity and mortality of lung cancer patients. We assessed whether aberrant expression of specific microRNAs could contribute to brain metastasis. Comparison of primary lung tumors and their matched metastatic brain disseminations identified shared patterns of several microRNAs, including common down-regulation of miR-145-5p. Down-regulation was attributed to methylation of miR-145's promoter and affiliated elevation of several protein targets, such as EGFR, OCT-4, MUC-1, c-MYC and, interestingly, tumor protein D52 (TPD52). In line with these observations, restored expression of miR-145-5p and selective depletion of individual targets markedly reduced *in vitro* and *in vivo* cancer cell migration. In aggregate, our results attribute to miR-145-5p and its direct targets pivotal roles in malignancy progression and in metastasis.

## INTRODUCTION

Almost one third of patients with systemic cancers develop brain metastases [1, 2]. In fact, brain metastases represent the most frequent occurring intracranial neoplasm in adult [2] and as such are a major cause of morbidity and mortality in patients with systemic cancers. The vast majority of brain metastases originate from primary carcinoma of either lung (40-50%) or breast (20-30%) as well as from melanoma (5-20%) [3, 4]. Currently, therapeutic approaches to treat brain metastases include surgery, whole-brain radiation therapy (WBRT), stereotactic radiosurgery (SRS), targeted-chemotherapy, or specific combinations of these treatments [5, 6]. Survival of patients with brain metastases typically ranges from 4-6 months, but might extend up to 12-24 months. This depends on diverse prognostic factors, such as age, general conditions, single or multiple brain metastases, co-occurrence of metastases at other sites, and the status and nature of the primary tumor [7].

MicroRNAs (miRs) are small non-coding RNAs of 18-22 nucleotides, which promote degradation and translational inhibition of imperfectly complementary target messenger RNAs [8]. MiRs are directly involved in the pathogenesis of many human cancers, including leukemia, lung, breast, brain, liver, colon, prostate and ovarian cancers [9, 10]. MiRs finely tune several cellular processes, including cell growth, apoptosis, differentiation, senescence, invasion and migration, and therefore might function as either tumor suppressors or oncogenes [11]. Altered miR expression leads to aberrant modulation of messenger RNAs whose encoded proteins can therefore undergo either up-regulation or down-regulation. Disregulation of miR expression in cancers can be ascribed to epigenetic changes, for instance aberrant DNA methylation and histone modification, and genetic alterations [12]. These can affect the synthesis of primary RNAs, miR processing/maturation and/or miR interaction with target mRNAs [13]. Many miRs are uniquely and differentially expressed in certain cancers compared to normal tissues, and miR expression profiles are currently considered as robust prognostic markers [9, 10, 14-17].

The present study focuses on miR profiles derived from brain metastases of human non-small-cell lung cancer (NSCLC). By applying our analyses to 29 patients we aimed at identifying a recurring signature characterizing metastatic brain lesions. Normal brain tissues (*N* = 6) collected from autopsies of patients deceased from causes other than lung cancer were used as control samples. In addition, to gain specificity we used brain metastases of melanoma or breast origin. Another comparator we utilized was normal lung tissue, collected from the peri-tumoral area of matched primary NSCLC lesions. These analyses uncovered consistent down-regulation of miR-145-5p expression in brain metastases, which we found to be caused by increased methylation of CpG islands in the 5′ regulatory region of miR-145-5p. As a consequence, the abundance of OCT-4 and EGFR, two validated targets of miR-145-5p, was increased in primary lung cancer and their matched-brain metastasis compared to non-tumoral tissues. Treatment of lung cancer cells with inhibitors of DNA methylation, such as 5-azacytidine and vorinostat, restored miR-145-5p levels and concomitantly reduced expression of oncoproteins encoded by miR-145-5p target mRNAs. Altogether these findings imply that miR-145-5p down-regulation enables up-regulation of a group of target proteins, whose coordinate activity contributes to brain metastasis.

## RESULTS

### Deregulated microRNA expression between primary lung cancers and brain metastases

**Figure 1:**
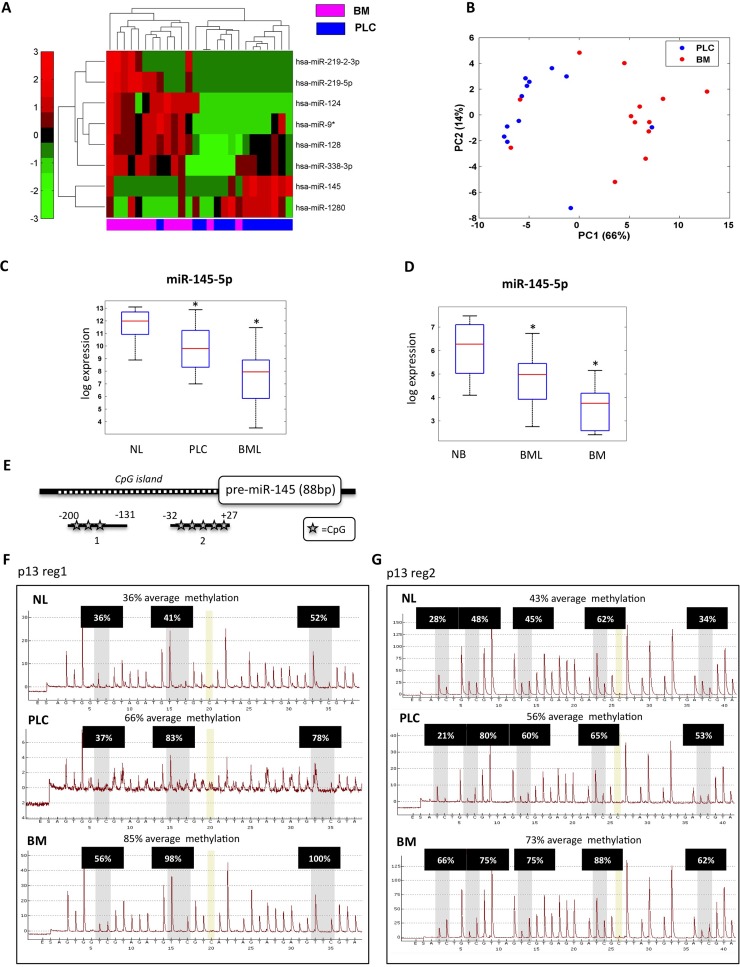
miR-145-5p expression is down-regulated in brain metastases **A.** Heat map of the identified signature of 8 miRs differentially expressed in 13 brain metastasis (BM) versus 13 matched primary lung cancer (PLC). **B.** Unsupervised principal component analysis (PCA). **C.** qRT-PCR analysis of miR-145-5p expression levels in 10 normal lung (NL), 13 matched primary lung cancer (PLC), and 29 brain metastases from lung (13 matched and 16 unmatched) (BML). **D.** qRT-PCR analysis of miR-145-5p expression levels in 6 normal brain (NB), 29 brain metastases from lung (BML) and 15 brain metastases from melanoma (6) and breast (9) (BM). **E.** Schematic representation of miR-145-5p CpG island genomic localization. **F.**-**G.** Pyrosequencing analysis of miR-145-5p CpG island methylation status in a representative patient of the casuistry (NL= normal lung; PLC= primitive lung cancer; BM= brain metastases; p= patient; reg1= region 1; reg2= region 2).

To explore the involvement of miRs in brain we collected FFPE (Formalin-Fixed, Paraffin-Embedded) samples from patients affected by one of the 3 main types of tumors exhibiting the highest incidence of brain metastases, namely melanoma, breast and lung cancer. [3, 4]. In particular, we focused on 13 primary lung cancers and their matched brain metastases; for 10 of these 13 samples we disposed also of the normal lung tissues. In addition, we collected 16 unmatched lung-derived brain metastases. Our collection also included 6 brain metastases from melanoma, 9 brain metastases from breast cancer and 6 non-tumoral brain tissues derived from autopsy (Table 1). We profiled the expression of 906 human miRs in 13 primary lung cancers and their matched brain metastases, and 2 non-tumoral brain tissues. These analyses identified 8 miRs that were differentially expressed between primary lung tumors and brain metastases (Figure 1A and Table 2). In particular, 6 miRs (miR-219-2-3p, miR-219-5p, miR-124, miR-9*, miR-128, miR-338-3p) were up-regulated, while miR-145-5p and miR-1280 were down-regulated in brain metastases compared to primary lung cancers. Unsupervised principal component analysis (PCA) showed that the expression levels of these 8 miRs discriminated the group of primary lung cancer samples from that of brain metastases (Figure 1B). The significance level of the difference between signal distributions of the eight selected miRs within the 26 analyzed samples was determined with supervised statistical test (Supplementary Figure S1A). To further evaluate the reliability of these results, we analyzed the expression levels of one up-regulated miR, miR-219-5p, and one down-regulated miR, miR-145-5p, in all 13 matched samples (primary lung cancer and matched brain metastases) by qRT-PCR (Supplementary Figure S1B). These experiments confirmed the results obtained by the array analysis.

**Table 1:** Casuistry description

FFPE samples	
normal brain (autopsy) *	6
normal lung (matched)	10
primary lung cancer *	13
brain metastases from lung (matched) *	13
brain metastases from lung	16
brain metastases from breast	9
brain metastases from melanoma	6

* samples profiled for microRNAs expression on Agilent platform

**Table 2:** miRs differentiating brain metastases from primary lung cancer tissues in 13 matched samples of the casuistry Differentiating miRs are listed with their p-values obtainded by paired t-test (pval) In the table are also indicated false discovery rate values (FDR), and folds of deregulation expressed in logaritmic scale (log2 fold).

miR	pval (Ttest paired)	FDR	log2 fold
hsa-miR-9*	0.0021	0.08	4.51
hsa-miR-124	0.0023	0.10	5.38
hsa-miR-128	0.0024	0.30	2.41
hsa-miR-145	0.0045	0.09	−1.75
hsa-miR-219-5p	0.0052	0.09	3.43
hsa-miR-338-3p	0.01	0.10	2.81
hsa-miR-219-2-3p	0.01	0.21	1.71
hsa-miR-1280	0.01	0.23	−2.26

### miR-145-5p expression is down-regulated in brain metastasis

We focused our attention on miR-145-5p, a well-known tumor suppressor miR, the expression of which is down-regulated in several tumor types compared to the respective normal tissue [18-22]. MiR-145-5p is an intergenic miR residing within a cluster that also includes miR-143-3p, another well-known tumor suppressor miR, as well as the complementary miRs miR-145-3p and miR-143-5p (Supplementary Figure S2A). Unlike previous analyses that reported similar expression patterns of miR-143-3p and miR-145-5p [23-25], we found that miR-143-3p expression was not co-regulated as shown by analysis of miR arrays (Supplementary Figure S2B). Similarly, no co-modulation of miR-143-5p and miR-145-3p was observed when comparing native tumors to brain metastases (Supplementary Figure S2B). Interestingly, miR-145-5p down-regulation was even stronger when comparing non-tumoral lung tissues and matched primary lung tumors (Figure 1C). Furthermore, down-regulation appeared to be specific to brain metastases, regardless of primary tumors tissue origin. Thus, miR-145-5p expression was down-regulated also in brain metastases originated from melanoma or from breast tumors when compared to normal brain (Figure 1D). Taken together, these findings might suggest that down-regulation of miR-145-5p is common in brain metastases, irrespective from the type of the originating primary tumor.

### CpG island methylation restrains the expression of miR-145-5p

To dissect molecular mechanisms underlying the decreased abundance of miR-145-5p in primary lung cancers and in brain metastases, we searched for potential epigenetic modifications affecting the regulatory regions of the miR-145 locus. To this end, we assessed the methylation status of a CpG island located 200 bp upstream to the genomic sequence encoding pre-miR-145-5p (Figure 1E). This was tested in normal lung tissue, the primitive lung cancer and brain metastases derived from 3 representative patients of our cohort. These analyses uncovered a significant increase in the methylation status of miR-145 CpG island when normal lung tissues were compared to primitive lung cancer and brain metastases (Figure 1F-1G and Supplementary Figure S3A-B). Congruently, methylated-DNA immunoprecipitation (meDIP), using an antibody specific to 5′methyl-cytosine revealed increased methylation of the analyzed CpG island (Supplementary Figure S3C). Altogether these findings propose that CpG island methylation within the miR-145 regulatory region contributes to down-regulation of mature miR-145-5p in brain metastases.

To further investigate the impact of methylation on the regulation of miR-145-5p expression we assessed the effects induced by two chemical epigenetic modifiers, namely 5-azacytidine (5-aza-Cd) and vorinostat (SAHA) on the abundance of miR-145-5p in 3 different metastatic cancer cell lines, namely H1299 (NSCLC), MDA-MB-231 (breast cancer) and M14-mel (melanoma). We found that both 5-aza-Cd and SAHA restored the expression of miR-145-5p in all three cell lines (Supplementary Figure S4A-B). This effect was reversible as the replenishment of the cell cultures with medium lacking SAHA caused down-regulation of miR-145-5p expression (Supplementary Figure S4C). In addition, 5-azacytidine and vorinostat induced the expression of pri-miR-145-5p, the miR-145-5p precursor (Supplementary Figure S4D), implying that methylation release of the miR-145-5p regulatory region enhanced transcription of downstream sequences. These findings paired with a reduction of the methylation status of the CpG island of the regulatory region of miR-145-5p upon treatment with 5-aza-Cd in the analyzed cancer cell lines (Supplementary Figure S4E and Supplementary Figure S5). Chromatin immunoprecipitation (ChIP) experiments revealed that SAHA treatment increased the acetylation status of the regulatory region of miR-145-5p, again supporting the possibility that the induction of mature miR-145-5p is due, at least in part, to the release of the inhibitory effect of methylation on chromatin conformation (Supplementary Figure S4F). Altogether these findings indicated that CpG island methylation within the miR-145 regulatory region contributes to the observed down-regulation of mature miR-145-5p in brain metastases.

### Ectopic expression of miR-145-5p restrains brain orthotopic tumor engraftment

**Figure 2:**
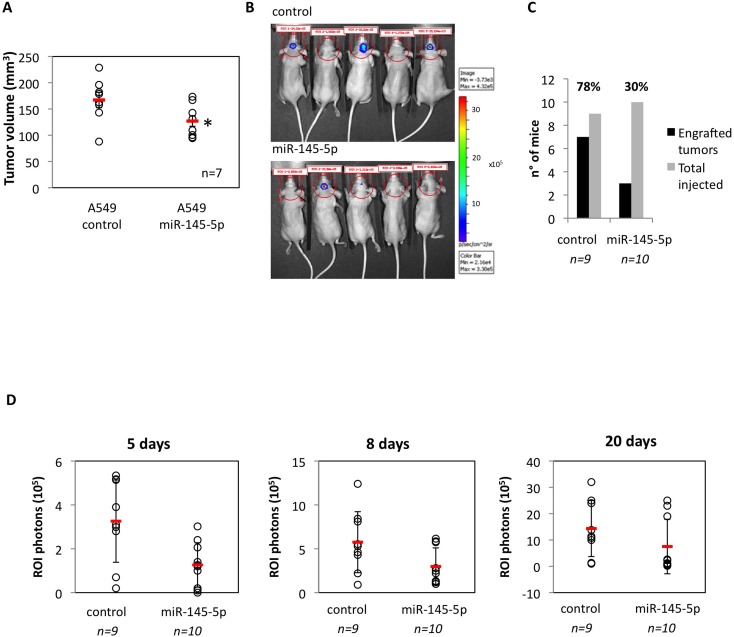
Restoration of miR-145-5p expression restrains *in vivo* and *in vitro* tumor invasion **A.** Tumor engraftment of A549 cells subcutaneously injected in 6-weeks old athymic nu/nu mice. **B.** Human non-small cell lung carcinoma A549-luc and the A549-miR145-5p cells were orthotopically injected below the dura. Tumor cell growth was monitored twice a week conducting quantitative bioluminescence imaging (qBLI) using the IVIS200 imaging station (Caliper Life Sciences), beginning one week after tumor cell injection. **C.** Numbers of engrafted tumors derived from A549-luc and A549-miR-145-5p cells respectively. **D.** Bioluminescence images were acquired at day 5, 8 and 20 after the orthotopical injectione with the IVIS Spectrum imaging system (PerkinElmer) and quantified by measurement of photon flux (photons/s/cm2/steradian) using the Living Image Software package (Perkin Elmer/Caliper Life Sciences).

To test *in vivo* the antitumoral effects of miR-145-5p, we subcutaneously injected nu/nu athymic mice with either human lung cancer cells, either control A549 cells, or A549 cells expressing an exogenous miR-145-5p (A549/miR-145-5p). We firstly observed *in vitro* that ectopic expression of miR-145-5p markedly reduced migration of A549 cells but had no effect on cell proliferation and viability (Supplementary Figure S6A-C). We found that the engraftment of A549/miR-145-5p cells was significantly less efficient than that of A549 control lung cancer cells (Figure 2A). To investigate the role of miR-145-5p on brain metastasis we performed intracranial orthotopically injection of A549-luc/miR-145-5p and A549-luc/control cells in nu/nu athymic mice. Bioluminescence images collected at day 8 and at day 20 revealed that A549-luc/miR-145-5p engrafted in 3/10 mice (30%) while A549-luc/control cells in 7/9 (64%) mice (Figure 2B-2D). Specifically, on day 5, three miR-145 mice displayed ROI >=2. On days 8 and 20, these three animals had median (range) ROI of 5.85 (5.75-6.15) and 23 (19-25) (Figure 2D). In contrast, on day 5, seven miR-145 mice had ROI<2. By day 8 or day 20, these seven animals had median (range) ROI of 1.32 (1-2.8) and 0.8 (0.2-2.6), respectively (Figure 2D). The median differences were statistically significant (*p* = 0.022 at day 8 and *p* = 0.017 at day 20). In parallel, amongst control mice, on day 5 seven animals had ROI >=2, and on day 8 and 20 these animals displayed median (range) ROI of 5.53 (4.3-12.4) and 13.8 (10-32) (Figure 2D), respectively. On day 5, the two control mice displayed ROI<2 and their median (range) on day 8 and day 20 were ROI of 1.55 (0.9-2.2) and 1.15 (1-1.3), respectively (Figure 2D). The median differences were statistically significant (*p* = 0.056 at day 8 and day 20). Altogether these findings strongly support an *in vivo* evidence of the role of miR-145-5p in brain metastasis.

### Ectopic expression of miR-145-5p impairs cell migration

**Figure 3:**
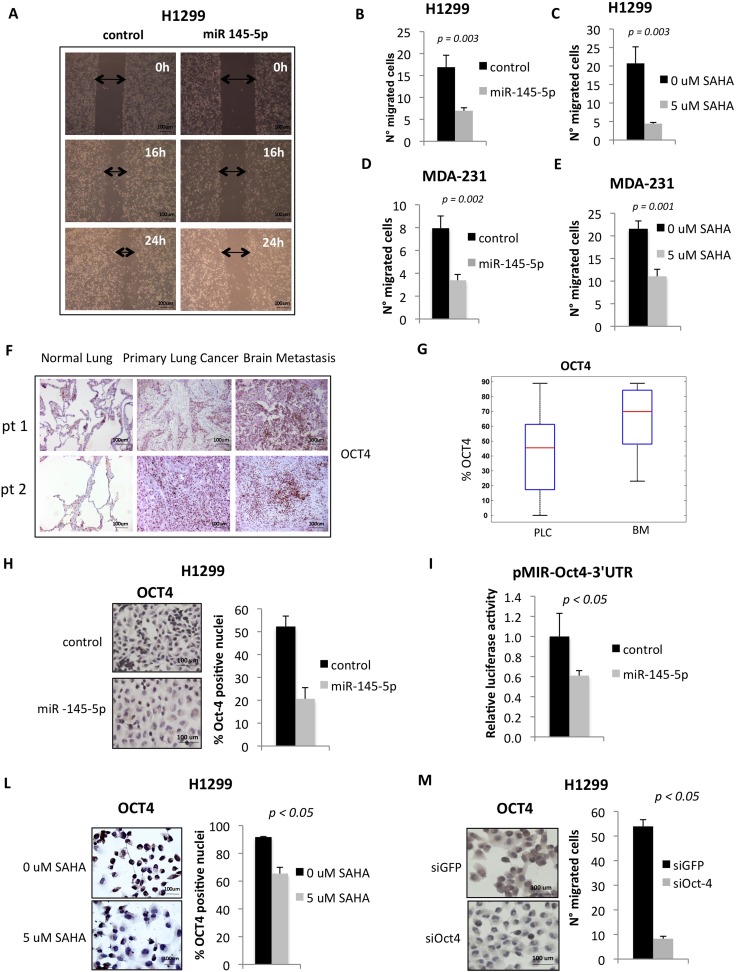
miR-145-5p expression impairs cell migration of H1299 and MDA-231 cells and inhibits Oct-4 protein **A.** Wound healing assay in H1299 cells upon miR-145-5p over-expression. **B.**-**E.** Transwell migration assay in H1299 and MDA-231 cells upon miR-145-5p over-expression **B.** and **D.** or 24 hours of 5 uM vorinostat (SAHA) treatment **C.** and **E. F.** Immunohistochemistry for Oct-4 in 2 representative patients slides (pt=patient). **G.** Percentage of positive Oct-4 staining in tissues (PLC= primary lung cancer; BM= brain metastases). **H.** Oct-4 immunocitochemistry in H1299 cells upon miR-145-5p over-expression. **I.** Renilla luciferase activity of Oct-4-3′UTR reporter gene in H1299 transiently transfected with miR-145-5p mimic or control mimic. **L.** Oct-4 immunocitochemistry in H1299 cells upon 24 hours of 5 uM vorinostat (SAHA) treatment. **M.** Transwell migration assay in H1299 cells upon Oct-4 RNA interference.

It has been previously reported that miR-145-5p exerts anti-tumoral effects ranging from growth arrest to inhibition of cell invasion [18, 26-29]. These effects are cell context dependent and occur through the selective targeting of specific mRNAs [30-33]. We found that ectopic expression of miR-145-5p markedly reduced migration of H1299 cells (Figure 3A-3B and Supplementary Figure S7A-B). On the contrary, miR-145-5p depletion determined an increase in cell migration (Supplementary Figure S7C). Interestingly, SAHA treatment of H1299 cells, which in part acts through the induction of miR-145-5p, impaired cell migration to a similar extent than that provoked by miR-145-5p ectopic expression (Figure 3C). No effects of miR-145-5p ectopic expression on H1299 cell proliferation were evidenced (Supplementary Figure S7D). Similar findings were observed upon ectopic expression of miR-145-5p or treatment of MDA-MB-231 cells with SAHA (Figure 3D-3E).

### Down-regulation of miR-145-5p releases the expression of OCT4, EGFR, MUC-1 and c-MYC proteins

Loss of tumor suppressor miRs is a frequent alteration in human tumors and leads to the heightened expression of proteins encoded by their target mRNAs. Indeed, we found that the expression of OCT-4 protein whose mRNA is a known target of miR-145-5p, was increased in both primary lung cancers and matched brain metastases when compared to matched normal lung tissues (Figure 3F-3G and Supplementary Figure S8) [30]. Ectopic expression of miR-145-5p in H1299 cells reduced OCT-4 protein expression (Figure 3H). This occurred through the direct binding of miR-145-5p to the 3′UTR of OCT-4 mRNA (Figure 3I). SAHA treatment, that induced miR-145-5p expression, reduced OCT-4 protein levels (Figure 3L). Interestingly, siRNA-mediated knock down of OCT-4 protein expression markedly reduced the migration of H1299 cells (Figure 3M).

**Figure 4:**
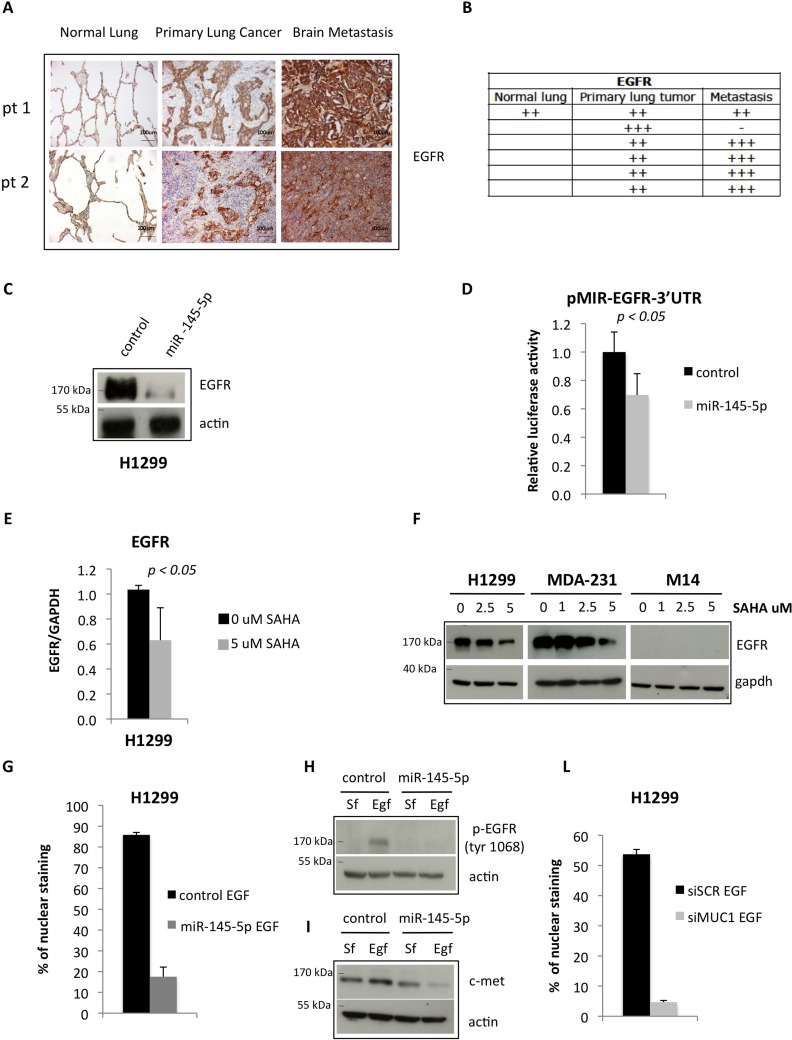
miR-145-5p impairs the expression of EGFR protein **A.** Immunohistochemistry for EGFR in 2 representative patients slides (pt=patient). **B.** Quantification of positive EGFR staining in tissues. **C.** Western-blot analysis of EGFR protein expression in H1299 cells upon miR-145-5p over-expression. **D.** Renilla luciferase activity of EGFR-3′UTR reporter gene in H1299 transiently transfected with miR-145-5p mimic or control mimic. **E.** qRT-PCR analysis of EGFR mRNA levels in H1299 cells upon 5 uM vorinostat (SAHA) treatment. **F.** Western-blot analysis of EGFR in H1299, MDA-231 and M14 cells upon 24 hours of vorinostat (SAHA) treatments. **G.** Quantification of immunofluorescence assay to analyze EGFR localization in H1299 cells treated with EGF (20 ng/mL) upon miR-145-5p over-expression. **H.** Analysis of pEGFR protein expression levels in H1299 cells treated with EGF (20 ng/mL) or maintained in serum free (Sf) medium upon miR-145-5p over-expression. **I.** c-Met protein levels in H1299 cells treated with EGF (20 ng/mL) or maintained in serum free (Sf) medium upon miR-145-5p over-expression. **L.** Quantification of immunofluorescence assay to analyze EGFR localization in H1299 cells treated with EGF (20 ng/mL) upon MUC-1 RNA interference.

**Figure 5:**
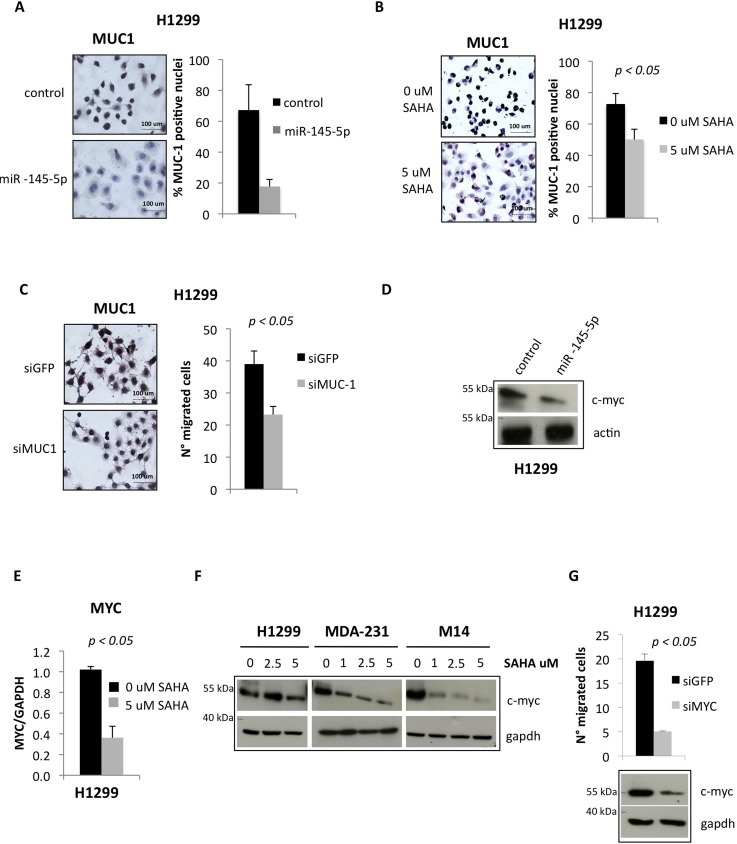
miR-145-5p impairs the expression of MUC-1 and MYC proteins **A.** MUC-1 immunocitochemistry in H1299 cells upon miR-145-5p over-expression. **B.** MUC-1 immunocitochemistry in H1299 cells upon 5 uM vorinostat (SAHA) treatment. **C.** MUC-1 immunocitochemistry in H1299 cells upon MUC-1 RNA interference (left panel) and transwell migration assay in the same conditions (right panel). **D.** Western-blot analysis of MYC protein expression levels in H1299 cells upon miR-145-5p over-expression. **E.** qRT-PCR analysis of MYC mRNA levels in H1299 cells upon 24 hours of 5 uM vorinostat (SAHA) treatment. **F.** Western-blot analysis of MYC in H1299, MDA-231 and M14 cells upon 24 hours of vorinostat (SAHA) treatments. **G.** MYC protein levels in H1299 cells upon MYC RNA interference and transwell migration assay in the same conditions.

Similar to OCT-4 protein, the expression of EGFR was increased (Figure 4A) in the same set of tissues analysed in Figure 3F (Figure 4A-4B). Ectopic expression of miR-145-5p reduced the expression of EGFR, while its depletion determined an increase EGFR in protein levels, and this occurred through the direct binding to the 3′UTR of EGFR mRNA (Figure 4C-4D and Supplementary Figure S10B) [31]. SAHA treatment reduced EGFR expression both the transcript and the protein levels (Figure 4E-4F). It has been shown that EGFR nuclear localization is enhanced by EGFR binding to MUC-1, with reduced expression of MUC-1 causing depletion of nuclear EGFR [34]. Since MUC-1 has been reported to be a target of miR-145-5p we aimed to assess whether its ectopic expression reduced MUC-1 protein level and impaired EGFR nuclear localization [32]. We found that ectopic expression of miR-145-5p markedly reduced MUC-1 expression (Figure 5A). Interestingly, this paired with marked reduction of EGFR nuclear localization, its tyrosine phosphorylation at residue 1068 and the induction of its downstream target MET (Figure 4G-4I and Supplementary Figure S9A). siRNA-mediated depletion of MUC-1 expression impaired EGFR nuclear localization (Figure 4L and Supplementary Figure S9B) [34]. Ectopic expression of miR-145-5p reduced the expression of two downstream effectors of MUC-1, such as β-catenin and Cyclin D1 (Supplementary Figure S10A). SAHA treatment reduced the expression of MUC-1 protein (Figure 5B). Similarly to SAHA treatment, siRNA-mediated knock down of MUC-1 expression impaired migration of H1299 cells (Figure 5C).

It has been previously shown that c-MYC is a target of miR-145-5p [33]. We found that ectopic expression of miR-145-5p reduced the expression of MYC protein while its depletion determined an increase in protein levels (Figure 5D and Supplementary Figure S10B). This occurred through the direct binding of either ectopically expressed or SAHA-induced expression of miR-145-5p to the 3′UTR of MYC mRNA (Supplementary Figure S10C-D). SAHA treatment reduced the expression of both MYC transcript and protein as a consequence of restoration of miR-145-5p expression (Figure 5E-5F). Depletion of c-MYC expression impaired migration of H1299 cells (Figure 5G).

Altogether these findings imply that miR-145-5p down-regulation releases aberrant expression of pro-invasive factors such as OCT-4, c-MYC, MUC-1 and instigates, directly as well as indirectly (i.e. via MUC-1) aberrant EGFR signaling (Table 3).

**Table 3:** miR-145-5p characterized targets with the relative references

gene target	reference
c-myc	Sachdeva et al., 2009 Breast cancer cells
EGFR	Cho WC et al., 2011 Lung cancer cells
MUC1	Sachdeva M and Mo Y Y, 2010 Breast cancer cells
Oct4	Yin et al., 2011 Lung cancer cells

### Tumor protein D52 (TPD52) is a novel target of miR-145-5p

**Figure 6:**
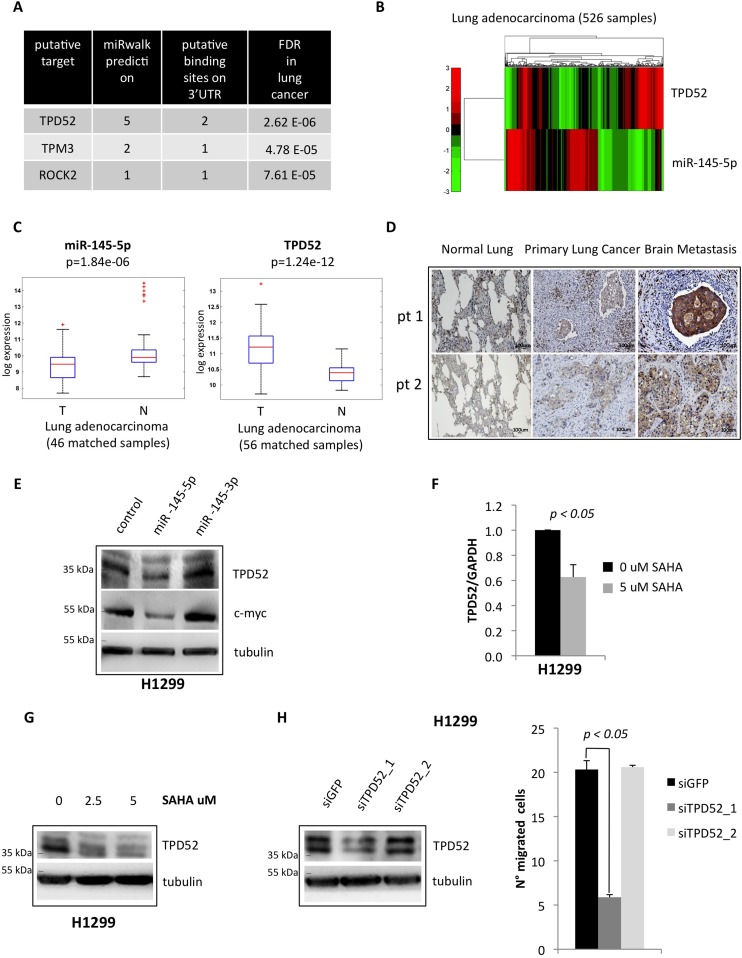
miR-145-5p impairs the expression of TPD52 protein **A.** List of the most significant miR-145-5p putative target genes with a known role in metastasis pathways, that resulted to be inversely correlated to miR-145-5p expression in the TCGA casuistry of 526 lung adenocarcinoma tissues. Genes were ranked by the number of the softwares that predicted miR-145-5p binding on the 3′UTR. **B.** Heat map of TPD52 and miR-145-5p genes expression in 526 tumoral samples of lung adenocarcinoma TCGA casuistry. **C.** miR-145-5p and TPD52 genes expression in matched samples of normal (N) and tumoral (T) tissues of lung adenocarcinoma TCGA casuistry. **D.** Immunohistochemistry for TPD52 in 2 representative patients slides (pt=patient). **E.** Western-blot analysis of TPD52 protein expression in H1299 cells upon miR-145-5p and miR-145-3p mimics over-expression. **F.** qRT-PCR analysis of TPD52 mRNA levels in H1299 cells upon 24 hours of 5 uM vorinostat (SAHA) treatment. **G.** Western-blot analysis of TPD52 in H1299 upon vorinostat (SAHA) treatments. **H.** TPD52 protein levels in H1299 cells upon TPD52 RNA interference (left panel) and transwell migration assay in the same conditions (right panel).

To further dissect the contribution of miR-145-5p down-regulation to metastasis we searched for additional, yet undiscovered, mRNA targets, the annotated functions of which could be related to dissemination of tumor cells. Putative mRNA targets of miR-145-5p were identified using miRWalk [35]. Next, permutation tests and false discovery procedures were used to further select out of the shortlisted miRs several mRNA targets that are deregulated when normal and tumoral samples are compared in The Cancer Genome Atlas (TCGA) lung adenocarcinoma data set (Figure 6A) [36]. Among these mRNAs, we noted TPD52, whose product promotes metastasis. Thus, TPD52 emerged as a potential and biologically relevant target of miR-145-5p in the process of metastatic colonization of brain [37, 38]. As shown in Figure 6B and Supplementary Figure S11A, TPD52 gene expression was found to be inversely correlated to miR-145-5p expression in both lung and breast cancer data sets from the TCGA consortium [36, 39]. Moreover in the lung data set we observed strong down-regulation of miR-145-5p and up-regulation of TPD52 mRNA when comparing tumoral and matched normal samples (Figure 6C). Indeed, the 3′-UTR of TPD52 contains two consensus binding sites miR-145-5p (Supplementary Figure S11B). Interestingly, TPD52 protein expression was increased in matched brain metastasis, when compared to both normal lung and primary lung cancer tissues (Figure 6D). In line with these observations, ectopic expression of miR-145-5p reduced the TPD52 protein levels (Figure 6E). This effect was not evidenced upon expression of miR-145-3p that is produced by the complementary strand of the miR-145 locus (Figure 6E). Interestingly, treatment of H1299 cells with vorinostat, which restored the expression of miR-145-5p, led to a reduction of TPD52 transcript and protein levels (Figure 6F-6G). At the functional level, siRNA-mediated knock down of TPD52 (siTPD52_1) markedly impaired migration of H1299 cells (Figure 6H), but siTPD52_2 or siGFP oligonucleotides, which failed to affect TPD52 expression, did not have any effect on migration of H1299 cells (Figure 6H).

**Figure 7:**
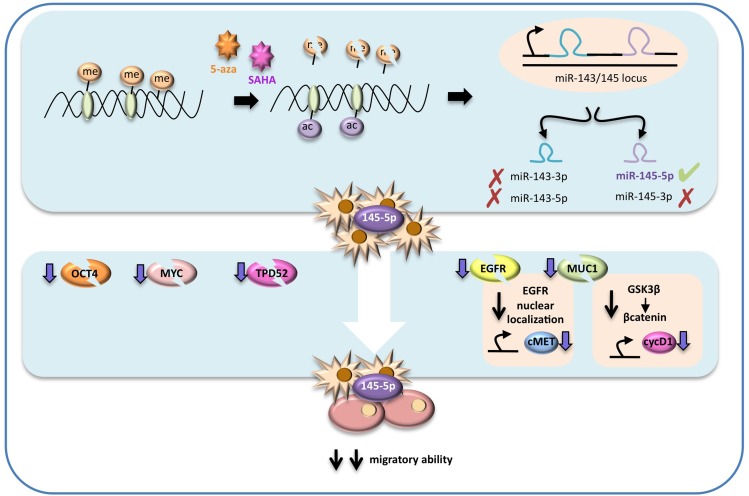
Schematic representation of the proposed molecular mechanism In highly metastatic lung cancer cells miR-145-5p expression is maintained at low levels by epigenetic modifications, such as hypermetylation and deacetylation of its promoter. Treatments with epigenetic drugs, such as vorinostat (SAHA) and 5-azacytidine (5-aza), could restore miR-145-5p expression, that in turns has a strong inhibitory effect on OCT-4, EGFR, MUC-1, MYC and TPD52 proteins expression. In particular MUC1 protein down-regulation potentiates the inhibitory effect on EGFR activity as it prevents EGFR nuclear localization. The final effect of miR-145-5p multi-targeting activity restoration results in a significant inhibition of the migratory ability of lung cancer.

Altogether, these findings identify TPD52 as a novel target of miR-145-5p. This implies that the aberrant expression of TPD52 due to loss of miR-145-5p activity might contribute to the metastatic process.

## DISCUSSION

This study identified a miR signature unique to brain metastases that originated in lung primary tumors. Previous work provided evidence supporting the ability of distinct miR signatures to differentiate between non-tumoral and tumoral tissues, as well as characterize specific subtype of disease [15-17, 20]. Adopting the newly identified miR signature, our study later focused on the miR-145 locus. This was motivated by previous reports that implicated the miR-145 locus in tumor suppression [18, 20, 26-29]. Unlike previous findings, which reported co-modulation of both chromosome 5 encoded miR-145 and miR-143, we found that expression of only miR-145-5p, but not miR-145-3p, miR-143-5p and miR-143-3p, was significantly different when comparing primary NSCLC lesions to their brain metastases. Down-regulation of both miR-145-3p and miR-145-5p has recently been reported as a long-term predictor of breast cancer using a case control-study nested in the ORDET prospective cohort study [19]. MiR-145-5p down-regulation was also reported in bone metastasis derived from primary prostate cancers [40]. MiR-145-5p down-regulation was also present, but significantly less pronounced, when comparing primary non-small lung cancers with matched non-cancerous lung tissues. In light of the results reported by Muti and colleagues it is likely that miR-145-5p down-regulation occurs early during tumorigenesis and it confers high metastatic potential. In this context, it is worthwhile mentioning that down-regulation of 24 miRs preceded and instigated massive up-regulation of oncogenic mRNAs in breast cancer cell lines, upon EGF stimulation. Collective down-regulation of this panel of miRs was also found when breast non-tumoral tissues were compared to matched tumoral tissues [41]. Along this vein, we found that miR-145-5p suppresses known target mRNAs, such as those encoding the potent oncoproteins OCT-4, EGFR, c-MYC, MUC-1 as well as the newly identified target TPD52. Accordingly, these mRNAs and proteins were up-regulated in both primary lung cancers and derived brain metastases, relative to non-tumoral lung tissues. Intriguingly, we previously showed that while miR-145-3p over-expression reduced proliferation of breast cancer cells, restoration of miR-145-5p activity impaired migration of breast cancer cell lines [19]. Indeed, we found that ectopic expression of miR-145-5p inhibited migration of lung adenocarcinoma cell lines without reducing cell proliferation. Similarly to the effect of miR-145-5p, selective knockdown of OCT-4, EGFR, MYC, MUC-1 and the newly identified TPD52 impaired migration of lung cancer cells. Currently, we poorly understand how miRs impact their target transcripts in a spatial and temporal specific manner in cancer. The mechanism might depend upon many factors, including cell context, affinity for and number of binding sites within the 3′UTRs of the target mRNAs, threshold of either up- or down-regulation of a specific miR, number of miRs that can simultaneously target a specific mRNA, miR location (intra- or intergenic) and the peculiarity of their respective genomic regulatory regions. Some of theses parameters might be more relevant than others when it comes to the regulation of a set of 5 important oncoproteins by miR-145-5p.

Epigenetic alterations on the regulatory region can also lead to aberrant expression of a specific miR [12]. Interestingly, we found that CpG island methylation at the miR-145 locus occurred in both primary lung cancers and, to a more pronounced extent, in matched brain metastases when compared to non-tumoral lung tissues. Restoration of miR-145-5p expression using either vorinostat or 5-azacytidine, which released methylation of the miR-145 locus, led to inhibition of migration of lung cancer cells and to down-regulation of OCT-4, MYC, EGFR, MUC-1 and TPD52 expression. Thus, it appears that selective down-regulation of miR-145-5p due to methylation of its regulatory regions might contribute, at least partially, to the establishment of a network of oncogenic proteins leading to metastasis. Strikingly, each of the verified miR-145-5p targets has been shown to play critical roles in migration, invasion and resistance to radio- and chemotherapy of advanced tumors [38, 42-45]. These features are shared by brain metastases, which are currently considered a major clinical issue in patients with advanced lung, breast, melanoma and renal cell cancer. Initial treatment of brain metastases typically includes radiotherapy, either whole brain radiotherapy (WBRT), stereotactic radiosurgery (SRS), or both. Surgical resection is generally reserved for good prognosis patients with limited extracranial metastases and a single brain lesion. For patients progressing through the up-front treatment, the therapeutic approach is quite variable and there are no standardised protocols. Targeted therapies, such as lapatinib, erlotinib, gefitininb and B-RAF inhibitors are increasingly used for the treatment of brain metastases from diverse solid tumors. A combined epigenetic therapy with 5-azacytidine and entinostat, inhibitors of DNA methylation and histone deacetylation, respectively, within a phase I/II trial in patients with recurrent metastatic NSCLC was well-tolerated and objective responses were observed [46]. Demethylation of four genes (*APC*, *RASSF1a*, *CDH13*, and *CDKN2a*) with negative prognostic impact on non-small lung cancer was observed in free plasma DNA of the 19 enrolled patients [46]. Altogether, these findings along the miRNA/mRNAs (miR-145-5p/EGFR-cMYC-MUC1-OCT4-TPD52) network here documented raise the possibility of, combining epigenetic drugs with conventional chemotherapy and oncogene-targeted therapeutics might hold great promises for rational-based therapeutic management of metastatic tumors.

## MATERIALS AND METHODS

### Cell cultures and treatments

Human cell lines H1299 and A549 were grown in RPMI medium (Invitrogen, Carlsbad, CA, USA) supplemented with 10% FBS, MDA-MB-231 cells and M14-mel cells were cultured in DMEM medium (Invitrogen-GIBCO, Carlsbad, CA, USA) with 10% (v/v) FBS; all cell lines were grown at 37°C in a balanced air humidified incubator with 5% CO2.

Cells were treated at the indicated concentration with vorinostat (SAHA), a histone deacetylase inhibitor, and with 5-azacytidine (5-aza-cd), a DNA methyltransferase inhibitor. MDA-MB-231 and M14-mel cells were harvested in colture for 96 hours and treated with 5-aza-cd after 24 hours and 72 hours. H1299 cells were maintained in colture for 96 hours and treated with 5-aza-cd every 24 hours for 3 days and with fresh medium without 5-aza-cd for the last 24 hours. For SAHA treatment, cells were plated and treated at the indicated concentration for 24h after which cells were harvested.

To analyse EGFR localization, H1299 cells were transfected with Pre-miRNA Precursor-Negative Control (Ambion) and Pre-miRNA145 (Ambion) at final concentration of 5nM and 48h after transfection the medium was replaced with RPMI without FBS. After 24h cells were treated with EGF (Sigma) at 20 ng/ml for 6h.

For evaluation of c-Met expression levels, H1299 cells were transfected with Pre-miRNA Precursor-Negative Control (Ambion) and Pre-miRNA145 (Ambion) at final concentration of 5nM and 48h after transfection the medium was replaced with RPMI without FBS. After 24h cells were treated with EGF (Sigma) at 20 ng/ml and 48h after treatment cells were harvested.

### Plasmids and transfections

For mature miR-145-5p expression, we used Pre-miRNA Precursor-Negative Control (Ambion) and Pre-miRNA145-5p (Ambion) at final concentration of 5nM. For miR-145-5p depletion we used Anti-miR miRNA Inhibitor Negative Control (Ambion) and Anti-miR has-miR-145-5p miRNA Inhibitor (Ambion) at final concentration of 10nM. H1299, MDA-MB-231 and M14-mel cells were transfected using Lipofectamine RNAiMAX (Invitrogen) according to the manufacturer's instructions.

For Luciferase assay H1299 cells were co-transfected in 24-well dishes using Lipofectamine 2000 (Invitrogen) with 200ng of OCT4-3′-UTR-(wt and mutant)-Luciferase vectors (a kind gift of Dr. Kenneth Kosik, UCSF Santa Barbara, CA, United States), 20 ng of the transfection control Renilla vector (phRLTK, Promega), and the pre-miRNA Precursor-Negative Control (Ambion) or the Pre-miRNA145-5p (Ambion) at the indicated concentrations.

Moreover H1299 cells were transfected in 24-well dishes using Lipofectamine 2000 (Invitrogen) with 25 ng of psiCHECK-2 reporter vector containing the 3′-UTR of the putative target gene Myc together with the pre-miRNA Precursor-Negative Control (Ambion) or the Pre-miRNA145 (Ambion) at the indicated concentration. Cells were harvested 48 hours post transfection and luciferase activities were analyzed by the dual-luciferase reporter assay system (Promega, Madison, WI) in the GloMax 96 Microplate Luminometer (Promega). Firefly luciferase was used to normalize the Renilla luciferase.

In addition H1299 cells were treated with 1 and 2,5 uM of SAHA for 24h to induce miR-145-5p expression and luciferase activity was tested as previously described.

For siRNA experiments, H1299 were transfected with siGFP (5′-AAGUUCAGCGUGUCCGGGGAG-3′), as control and siMYC (5′-GCCACAGCAUACAUCCUGU-3′) at 400 pmoli, siOCT4 (AAGGAUGUGGUCCGAGUGUGG), siMUC1 (5′-AAGACUGAUGCCAGUAGCACU-3′), siTPD52_1(GCGGAAACUUGGAAUCAAU) and siTPD52_2 (GGAGAAGUCUUGAAUUCGG) at 600 pmoli (Eurofins, MWG, Operon) for 48 hours.

### Study population

The cohort of paraffin-embedded tissues (FFPE) used for array validation consisted of 13 serial matched samples, primary lung cancers (PLC) and brain metastases (BML) plus 16 brain metastases from lung cancer (BML) and 2 normal brains (NB). Four additional cases of normal brain (NB), 6 brain metastases from melanoma and 9 brain metastases from breast cancer (BM) were recruited for qRT-PCR analysis of miR-145 expression levels. Following excision, fresh tissue samples were immediately frozen in liquid nitrogen and stored at −80°C until RNA extraction. All tissue samples used throughout the study were obtained from patients from Italian National Cancer Institute ‘Regina Elena’, Rome, Italy. The ethical committee of the institute has approved the study.

### RNA extraction, labeling and microarray hybridization

RNA from FFPE samples was extracted using the miRneasy FFPE kit (QIAGEN) following the manufacturer's instructions. The concentration and purity of total RNA were assessed using a Nanodrop TM 1000 spectrophotometer (Nanodrop Technologies, Wilmington, DE, USA). Total RNA (100 ng) was labeled and hybridized to Human miRNA Microarray Rel 14 V2 (Agilent). Scanning and image analysis were performed using the Agilent DNA Microarray Scanner (P/N G2565BA) equipped with extended dynamic range (XDR) software according to the Agilent miRNA Microarray System with miRNA Complete Labeling and Hyb Kit Protocol manual. Feature Extraction Software (Version 10.5) was used for data extraction from raw microarray image files using the miRNA_105_Dec08 FE protocol.

### Total RNA extraction from cells and reverse transcriptase

Total RNA was extracted using the TRIZOL Reagents (GIBCO). One microgram of total RNA was reverse-transcribed at 37°C for 60 minute in the presence of random hexamers and Moloney murine leukemia virus reverse transcriptase (Invitrogen). Specific oligonucleotides for the genes listed in Supplementary Table 1 and Supplementary Table were used for PCR analyses. GAPDH, TPD52, MYC and EGFR genes expressions were measured by real-time PCR using the Sybr Green assay (Applied Biosystems, Carlsbad, CA, USA) on a StepOne instrument (Applied Biosystems).

Small amount of RNA (10 ng) was reverse-transcribed using the TaqMan microRNA Reverse Transcription Kit (Applied Biosystem) and Real time-PCR of miR expression was carried out in a final volume of 10 ul using ABI Prism 7000 Sequence Detection System (Applied Biosystems). The PCR Reactions were initiated with a 10 minute incubation at 95°C followed by 40 cycles of 95°C for 15 seconds and 60°C for 60 seconds. RTq-PCR quantification of miR expression was performed using TaqMan MicroRNA® Assays (Applied Biosystems) according to the manufacturer's protocol. RNU19 was used as endogenous control to normalize miR expression. All reactions were performed in duplicate.

For measurement of the precursor miR expression, PCR was performed using 2X red Mix DNA Polymerase MasterMix (RBC Bioscence) using the primers listed in Supplementary Table 1. PCR was performed at 95°C for 5 min, 35 cycles at 95°C for 30 s, 60°C for 40 s and 72°C for 40 s followed by a final extension at 72°C for 10 min. The PCR products were loaded on 2% agarose gel for analysis.

### Lysate preparation and immunoblotting analysis

Cells were lysed in buffer with 50 mM Tris-HCl pH 8, with 1% NP-40 (Igepal AC-630) 150mM NaCl, 5mM EDTA and fresh protease inhibitors. Extracts were sonicated for 10 s and centrifuged at 12000 ×rpm for 10 min to remove cell debris. Protein concentrations were determined by colorimetric assay (Bio-Rad).

Western blotting was performed using the following primary antibodies: mouse monoclonal anti-Gapdh (Santa Cruz Biotechnology, Santa Cruz, CA, USA), mouse monoclonal anti-Cyclin D1 (Invitrogen), mouse monoclonal anti-Oct4 C-10 (Santa Cruz Biotechnology sc-5279), rabbit monoclonal anti-Egfr (Cell Signaling Tecnology, C74B9), rabbit polyclonal anti Phospho-Egfr (Tyr1068) (Santa Cruz Biotechnology), mouse monoclonal anti-Met (25H2) (Santa Cruz Biotechnology), hamster monoclonal anti-Muc1 (Ab-5) (Thermoscientific), rabbit monoclonal anti-c-Myc (Cell Signaling D84C12-XP), mouse monoclonal anti B-actin (ACTBD11B7) (Santa Cruz Biotechnology sc-81178), rabbit polyclonal anti-TPD52 H-54 (Santa Cruz Biotechnology sc-67063).

Secondary antibodies used were goat anti-mouse, goat anti-rabbit, conjugated to horseradish peroxidase (Amersham Biosciences, Piscataway, NJ, USA) and goat anti-Armenian hamster conjugated to horseradish peroxidase (Santa Cruz Biotechnology sc-2443). Immunostained bands were detected by chemiluminescent method (Pierce, Rockford, IL, USA).

### Cell cycle analysis

Samples were collected over the indicated time points and fixed in 70% ethanol overnight. Fixed cells were treated with RNase for 20 min before addition of 5 mg/ml PI and analyzed by FACS.

### Wound healing assay

H1299 cell lines transfected with pre-miRNA Precursor-Negative Control (Ambion) and the Pre-miRNA145-5p (Ambion), were grown to 80% confluence in 6-well tissue culture plates and wounded with a sterile 200 ul pipet tip to remove cells by perpendicular linear scrapes. PBS 1x washing was used to remove loosely attached cells. The cells were incubated in full medium with 10% FBS for 24 h. The progression of migration was photographed immediately, at 16 and at 24 h after wounding.

### Transwell migration assay

Migration assay was performed using a 24-well plate with a non-coated 8-mm pore size filter in the insert chamber (BD Falcon, Franklin Lakes, NJ, USA). Cells were transfected with pre-miRNA Precursor-Negative Control (Ambion) and the Pre-miRNA145 (Ambion), or with Anti-miR miRNA Inhibitor Negative Control (Ambion) and Anti-miR has-miR-145-5p miRNA Inhibitor (Ambion), or treated with SAHA at 0-5 uM, or silenced with siMyc at 400 pmoli or siOct4, siMuc1, siTPD52_1 and siTPD52_2 at 600 pmoli (Eurofins, MWG, Operon). After 48 hours from transfection or treatment, cells were resuspended in DMEM or RPMI media without FBS and seeded into the insert chamber. Cells were allowed to migrate for 12 h into the bottom chamber containing 0,7 ml DMEM or RPMI media containing 5% FBS in a humidified incubator at 37°C in 5% CO2. Migrated cells which had attached to the outside of the filter were visualized by staining with DAPI and counted.

### Immunofluorescence and immunocytochemistry

For immunocytochemistry assay cells were seeded onto glass coverslips (Marienfeld laboratory glassware, Lauda-Ko¨nigshofen, Germany) at 4×10^4^ cells/well into 6-well dishes (COSTAR), transfected with Pre-miRNA Precursor-Negative Control (Ambion) and Pre-miRNA145-5p (Ambion) at final concentration of 5nM or silenced for the target protein and fixed 48 h later with 4% formaldehyde in PBS for 15 min at room temperature (RT). Cells were permeabilized with 1% Triton X-100 in PBS for 10 min. After washing with PBS, the coverslips were incubated with Oct-4 antibody C-10 (Santa Cruz Biotechnologysc-5279) and Muc1 antibody (Ab-5) (Thermoscientific) diluted in 5% BSA/PBS for 1 h at RT. Cells were incubated with peroxidase inhibitor before primary antibody incubation. Protein staining was revealed through DAB enzymatic reaction while nuclei were counterstained with hematoxylin.

For immunofluorescence assay, 48h after transfection the media was replaced with RPMI without FBS. The day after cells were treated with Egf (Sigma) at 20 ng/ml for 6h and fixed with 4% formaldehyde in PBS. Then cells were washed twice with 0.02% Tween-20 and 1% BSA in PBS, followed by incubation with Alexa Flour 488 (rabbit) conjugated secondary antibodies (Molecular Probes Inc., Eugene, OR, USA) for 30 min at RT. After washing three times with 0.02% Tween-20 and 1% BSA in PBS, the coverslips were counterstained with DAPI 5 min and mounted with Vectashield (Vector Labs, Burlingame, CA, USA). Cells were examined under a Zeiss LSM 510 laser scanning fluorescence confocal microscope (Zeiss, Wetzlar, Germany).

### Immunohystochemistry

Formalin-fixed and paraffin-embedded 5 μm sections were stained with haematoxylin and eosin or stained with anti-Oct-4 antibody (SC-5279, Santa Cruz Biotechnology, 1:50), anti-EGFR antibody (Cell Signaling Tecnology, C74B9, 1:50), anti-TPD52 H-54 (SC-67063, Santa Cruz Biotechnology, 1:100) using BENCHMARK ULTRA VENTANA (Roche, Tucson, AZ, USA). Seven randomly chosen fields from each sample were scored.

### Methylated DNA immunoprecipitation assay (MeDIP)

Genomic DNA of one patient was isolated using the QIAamp DNA FFPE Tissue Kit (Qiagen, Hilden, Germany) and the resulting DNA was quantified on a Nanodrop spectrophotometer. Immunoprecipitation of methylated DNA was prepared as Weber et al, 2005; the antibody against 5-methyl-cytosine used for immunoprecipitation was from Abcam ab1884, San Diego, CA.

qRT-PCR reactions were carried out in duplicate on specific genomic regions using TaqMan Master Mix (Applied Biosystem). The list of primers used for amplification on methylated region is summarized in Supplementary Table 3. The resulting signals were normalized for primer efficiency by carrying out qRT-PCR for each primer pair using Input DNA.

### DNA isolation, sodium bisulfite conversion and pyrosequencing analysis

Genomic DNA was isolated using the QIAamp DNA FFPE Tissue Kit (Qiagen, Hilden, Germany). Sodium bisulfite modification of 800 ng DNA was performed using the DNA Methylation kit (Diatech Pharmacogenetics) according to the manufacturer's protocol. Modified DNA was subjected to PCR amplification of the specific promoter region of pri-miR-145 using the Corbett Life Science Rotor-Gene™ 6000. The primer sequences are listed in Supplementary Table 4. The resulting PCR products were analyzed by gel electrophoresis to confirm the size of the product and rule out the formation of primer dimers. The specific PCR products were then subjected to quantitative pyrosequencing analysis using a PyroMark Q96 ID (Qiagen) according to the manufacturer's protocol. The genomic location of the bisulfite pyrosequencing assays and the number of CpG sites investigated in each assay are shown in Figure 1. The pyrosequencing analysis was performed with PyroMarker CpG software 1.0.11 (Qiagen).

### Formaldehyde cross-linking and chromatin immunoprecipitation

H1299, MDA-MB-231 and M14-mel cells were treated with SAHA at the indicated concentration. After 24h formaldehyde cross-linking and chromatin immunoprecipitations were performed as described (Di Agostino et al., 2006). The chromatin was immunoprecipitated with anti-acetyl-Histone H4 antibody (Millipore) or no antibody as negative control. Primers used for the amplification by qRT-PCR of the different regulatory regions of miR-145-5p are listed in Supplementary Table 5.

### Cell proliferation assay

A549 cells were seeded into six-well dishes and transfected in triplicated as indicated. 6×10^4^ cells were seeded for this assay. Cells were collected and manual counted at 0, 24, 48, 72 hours after transfection.

### Viability assay

Cell viability was evaluated using the ATPlite™ Luminescence Assay System and following the manufacturer's instructions. Luminescence was read by the EnSpire^®^ Multimode Plate Reader (PerkinElmer, Whaltman, MA, USA).

### *In vivo* A549 cells subcutaneous tumor engraftment

All studies have been performed in accordance with “Directive 86/609/EEC on the protection of animals used for experimental and other specific purposes” and made effective in Italy by the legislative Decree DL 116/92.

14 athymic nu/nu mice 6-weeks old (Harlan) were utilized. After 1 week of acclimation they were housed four to a plastic cage and fed on basal diet (4RF24, Mucedola S.r.l.) with water ad libitum, in an animal facility controlled at a temperature of 23 ± 2°C, 60 ± 5% humidity, and with a 12 h light and dark cycle. Before injection, cells were washed once in PBS and then the pellet was re-suspended in 50% RGF matrigel (BD Biosciences) solution in Medium 199 and injected in the right flank of the mice in 200μl volume/mouse. 2×10^6^ A549 cells/mouse were injected for a total of 7 mice/group. Tumor growth was monitored weekly by caliper measurement and tumor volume was determined by the formula (D x d^2^)/2.

### Establishment of orthotopic brain tumor xenografts in mice

Human non small cell lung carcinoma A549-luc and the A549-miR-145-5p cell lines were collected by trypsinization, washed in PBS 1X, centrifuged at 1200 rpm x 10 mins at RT, re-suspended in PBS 1X w/o Ca^++^/Mg^++^ at the density of 2×10^5^ cells/10 μl (2×10^7^ cell/ml) and maintained on ice until the *in vivo* injection. Stereotaxic (Stoelting stereotaxic apparatus) tumor cell injection was performed in 20 Athymic nude mice 6 week-old (Harlan). Under deep anaesthesia (Zoletil/Rompum, 80 mg/kg +10 mg/kg) a 2 mm hole was drilled in the skull at the level of bregma and the cells suspension was injected into mouse brain using a glass micropipette equipped with 26 gauge needle. Cells were injected setting the stereotaxic coordinates to 5 mm below the dura. The solution was slowly injected over 4 min and the needle was left in place for an additional 1 min. The needle was then slowly withdrawn and the incision closed. Tumor cell growth was monitored twice a week conducting quantitative bioluminescence imaging (qBLI) using the IVIS200 imaging station (Caliper Life Sciences), beginning one week after tumor cell injection.

### Bioinformatics analysis

Array analysis was performed using Matlab (The MathWorks Inc.). Signals were extracted using Agilent Feature Extraction, quantile normalized and log2-trasformed. Paired and unpaired T-test were applied to evaluate significantly deregulated miRNAs. For signature selection we considered as significant pvalues less than 0.01. A False Discovery Rate procedure (Storey, 2002) for multiple comparisons was also included in the analysis. Hierarchical Clustering and Principal Component Analysis were used to evaluate the efficacy of the selected signature.

## SUPPLEMENTARY MATERIAL FIGURES AND TABLES


