# Supplementary material for: Epigenetic silencing of miR-145-5p contributes to brain metastasis
Source: Oncotarget. 2015 Sep 30;6(34):35183–201. doi: 10.18632/oncotarget.5930 (PMC4742098; doi:10.18632/oncotarget.5930)
Supplement: Supplementary file 1 [file oncotarget-06-35183-s001.pdf]

Epigenetic silencing of miR-145-5p contributes to brain metastasis

Supplementary Material

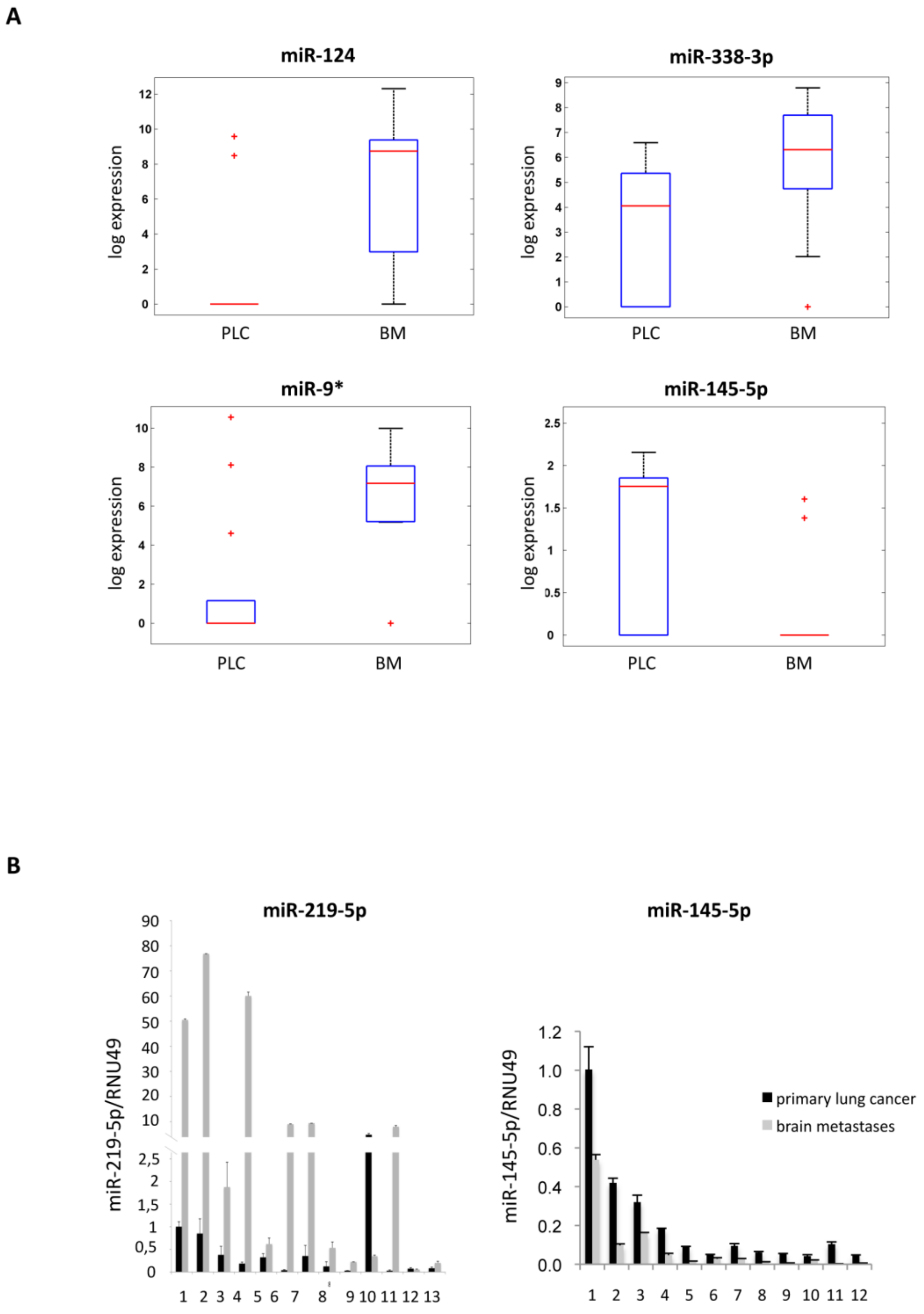

Supplementary figure S1.

A) Supervised statistical test of the significance level of the difference between signal distributions of 4 miRs between the 8 miRs of the signature within the 13 matched samples analyzed (PLC= primary lung cancer; BM= brain metastases). **B)** qRT-PCR validation of 2 miRs of the signature.

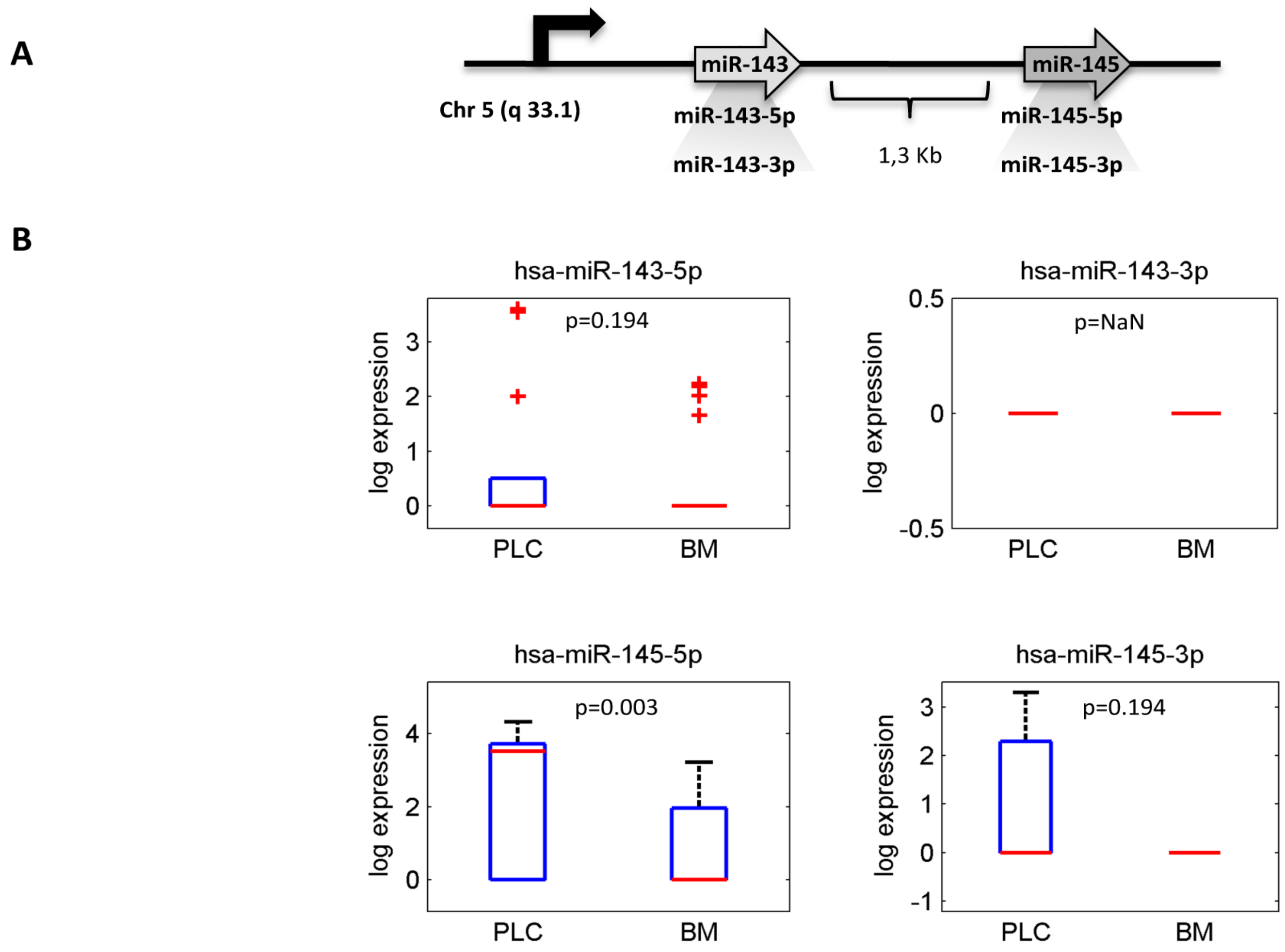

Supplementary figure S2.

A) Schematic representation of miR-143/miR-145 cluster. **B**) Microarray signal distributions of miR-143-5p, miR-143-3p, miR-145-5p and miR-145-3p.

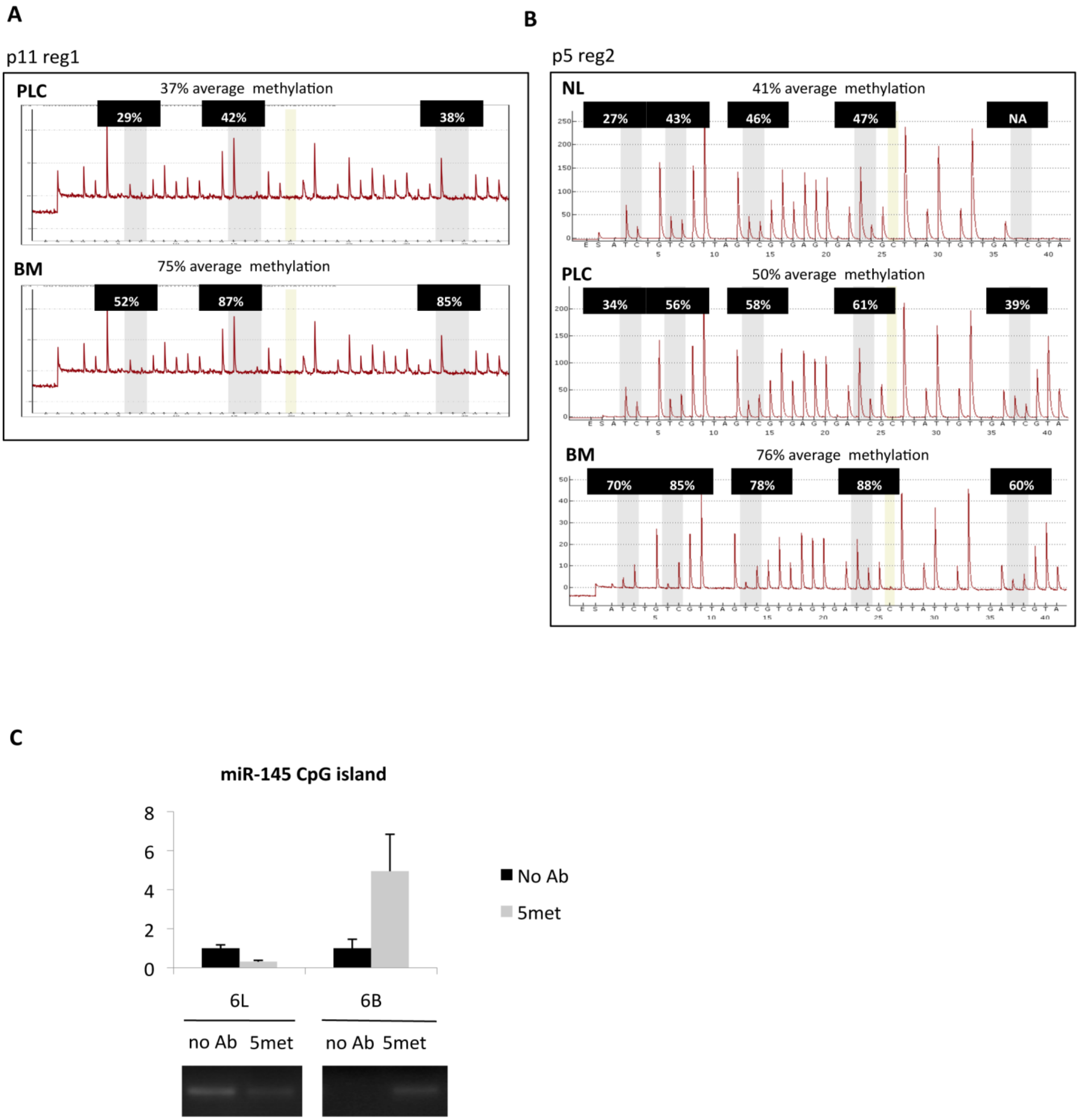

Supplementary figure S3.

A-B) Pyrosequencing analysis of miR-145-5p CpG island methylation status in 2 representative patients of the casuistry (NL= normal lung; PLC= primitive lung cancer; BM= brain metastases; p= patient; reg1= region 1; reg2= region 2). **C)** meDIP analysis of miR-145-5p gene CpG island methylation status in patient 6 (6L= primary lung cancer; 6B= brain metastases).

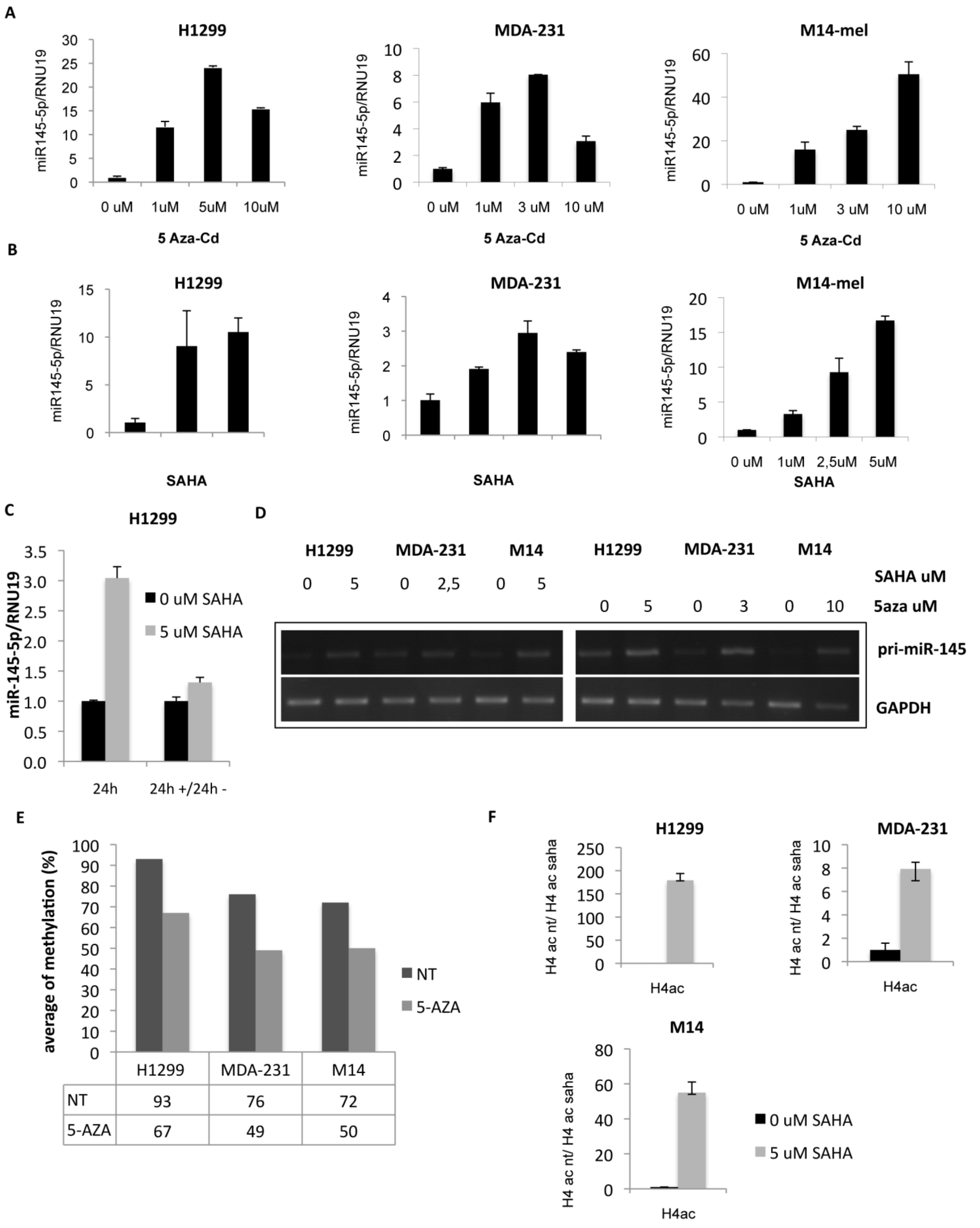
A
H1299
MDA-231
M14-mel
miR145-5p/RNU19
0 uM
1uM
5uM
10uM
1 uM
3 uM
10 uM
5 Aza-Cd
B
SAHA
0 uM
1uM
2,5uM
5uM
C
H1299
miR-145-5p/RNU19
0 uM SAHA
5 uM SAHA
24h
24h +/24h -
D
H1299
MDA-231
M14
SAHA uM
5aza uM
0
5
2,5
3
10
pri-miR-145
GAPDH
E
average of methylation (%)
NT
5-AZA
H1299
MDA-231
M14
NT 93 76 72
5-AZA 67 49 50
F
H1299
MDA-231
M14
H4 ac nt/ H4 ac saha
H4ac
0 uM SAHA
5 uM SAHA

Supplementary figure S4.

A-B) qRT-PCR of miR-145-5p expression levels in 3 different cell lines representative of the casuistry (H1299, MDA-231, M14) upon 5-azacytidine (5-aza-cd) and vorinostat (SAHA) treatments at the indicated doses. **C)** qRT-PCR of miR-145-5p expression levels in H1299 cells upon 24 hours of 5uM vorinostat (SAHA) treatment and after 24 hours without SAHA in the medium **D)** RT-PCR analysis of pre-miR-145-5p expression levels in 3 different cell lines representative of the casuistry (H1299, MDA-231, M14) upon 5-azacytidine (5-aza-cd) and vorinostat (SAHA) treatments at the indicated doses. **E)** Pyrosequencing analysis of miR-145-5p CpG island methylation status in cancer cell lines treated with 5-azacytidine . **F)** ChIP analysis of acetylated histone H4 occupancy on miR-145-5p regulatory regions in 3 different cancer cell lines upon treatment with 5uM SAHA.

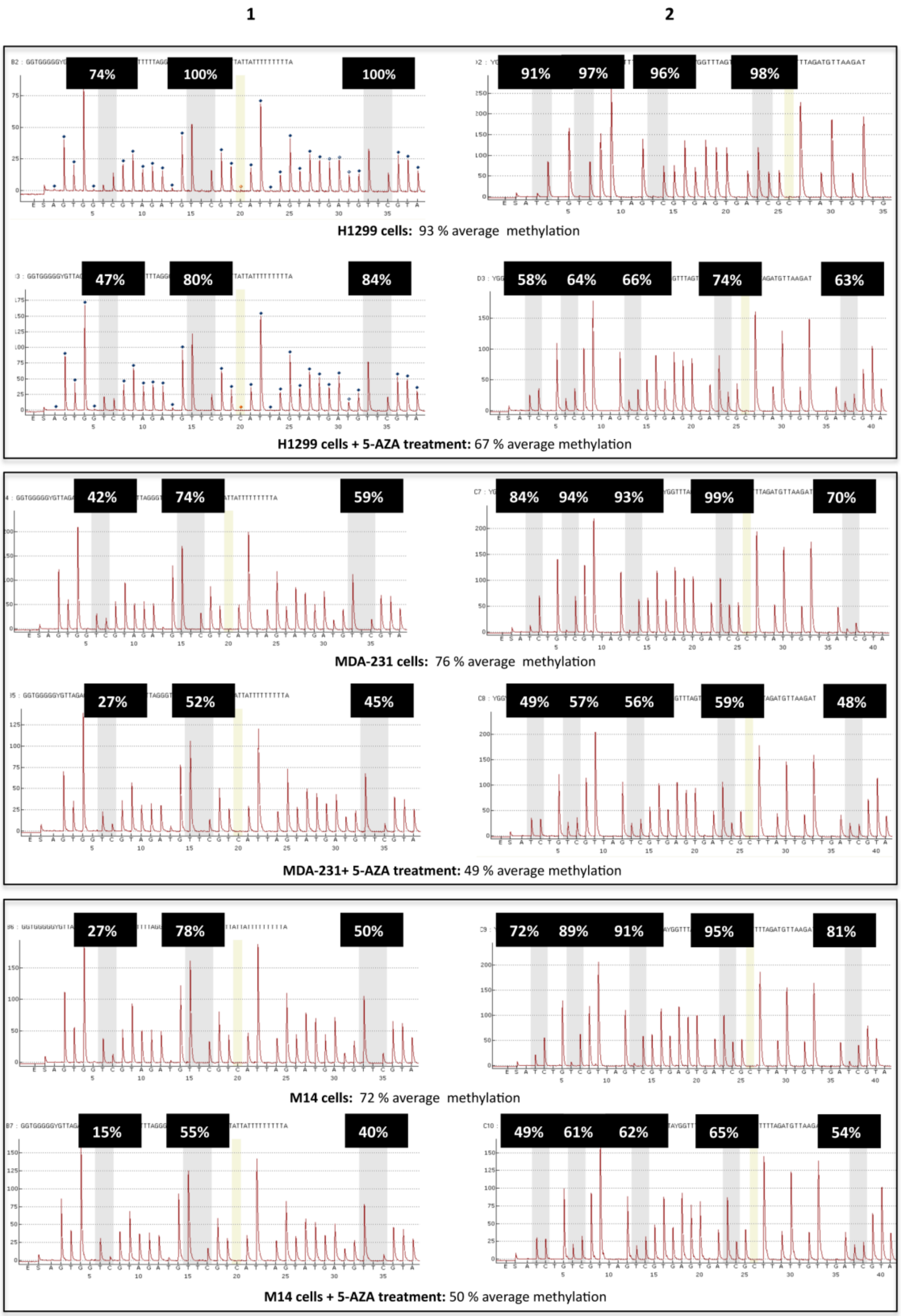

Supplementary figure S5.

Pyrosequencing analysis of miR-145-5p CpG islands 1 and 2 methylation status in 3 cancer cell lines representative of the casuistry (H1299, MDA-231 and M14) treated with 5 uM 5-azacytidine (1= region 1; 2= region 2).

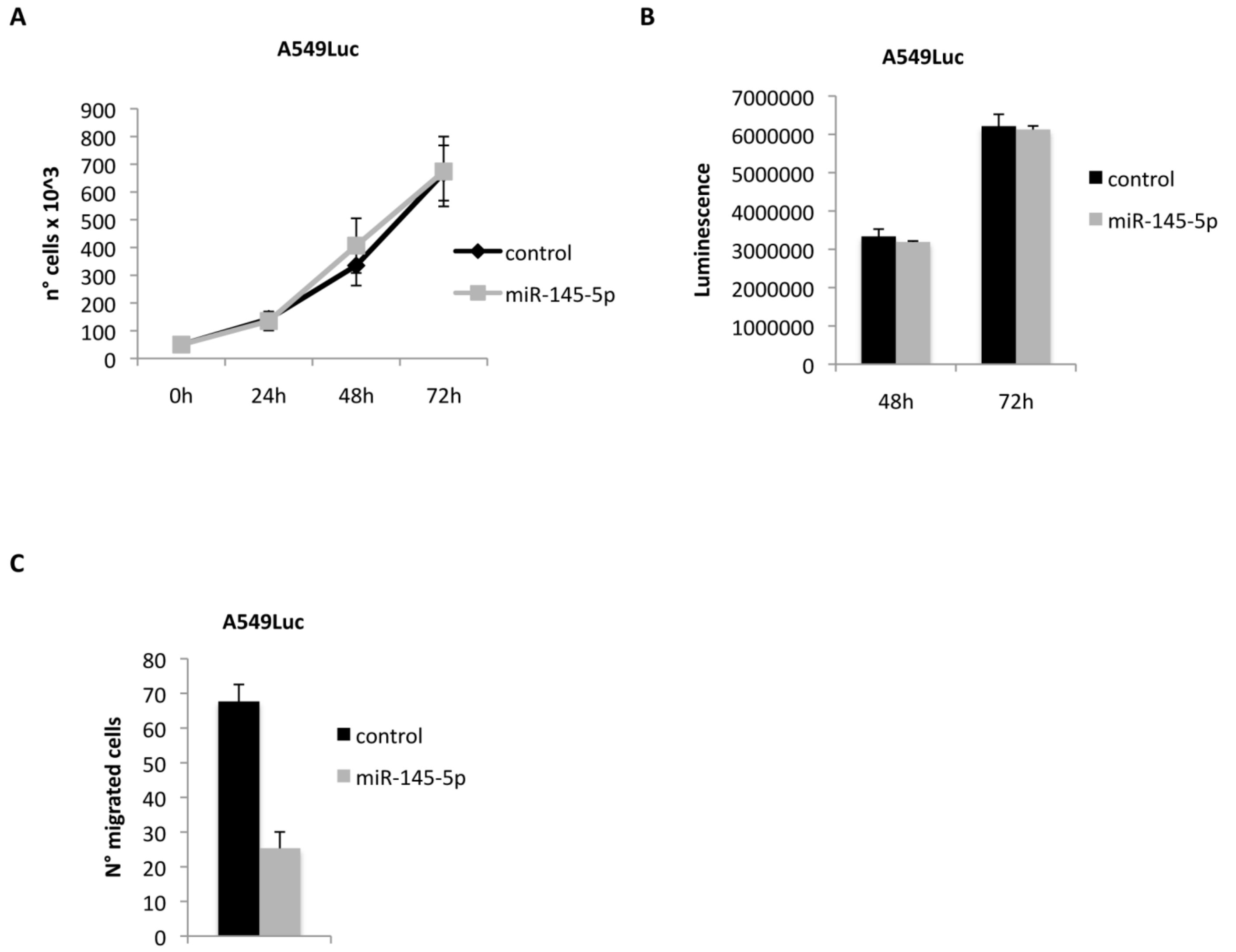

Supplementary figure S6.

A) Proliferation assay in A549 cells transiently transfected with miR-145-5p mimic or control mimic. **B)** Viability assay in A549 cells transiently transfected with miR-145-5p mimic or control mimic. **C)** Transwell migration assay in A549 cells upon miR-145-5p over-expression.

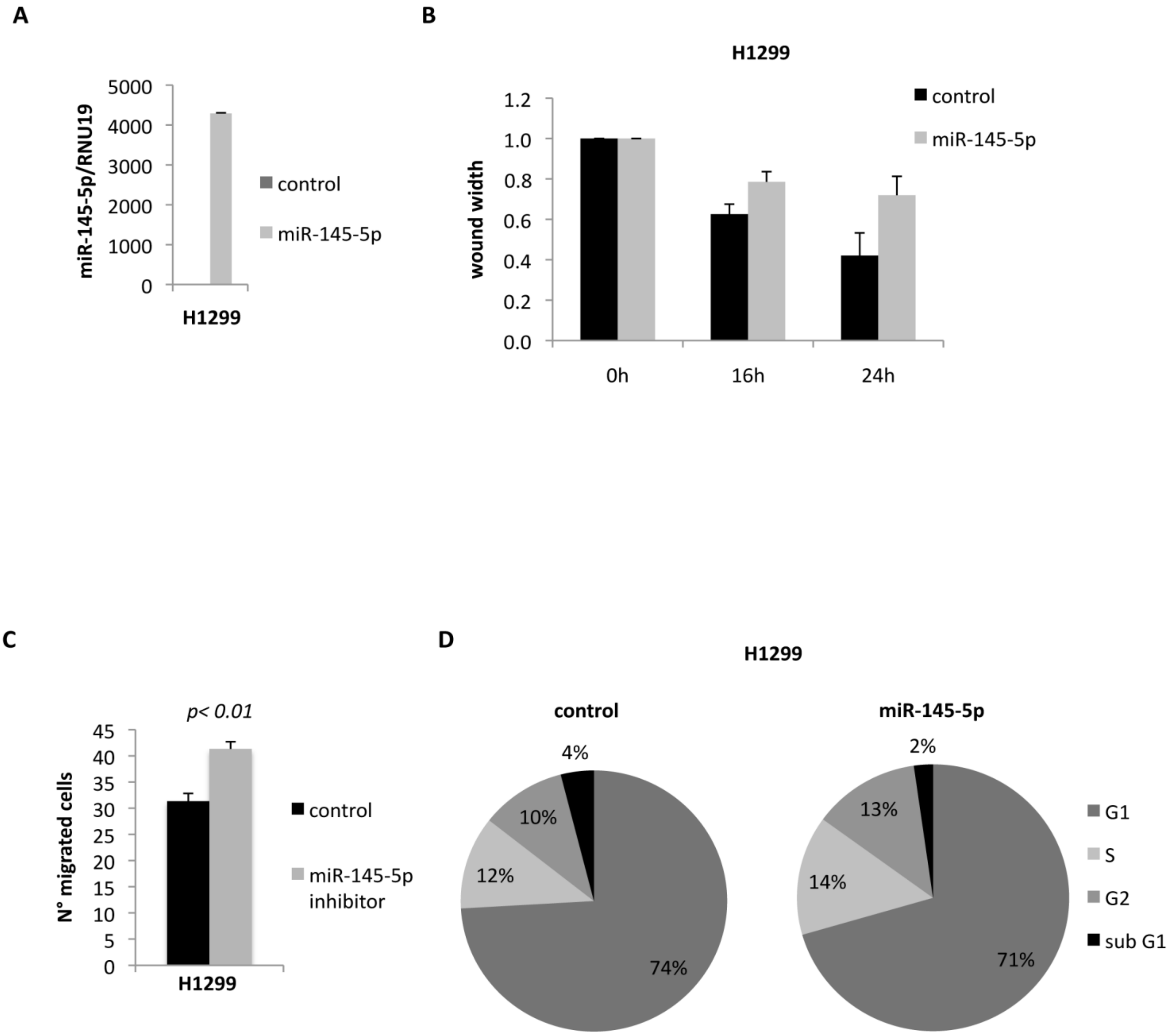

Supplementary figure S7.

A) qRT-PCR analysis of miR-145-5p expression levels in H1299 cells transiently transfected with miR-145-5p mimic or control mimic. **B)** Quantification of wound healing assay in H1299 cells upon miR-145-5p over-expression. **C)** Transwell migration assay in H1299 cells upon miR-145-5p depletion. **D)** FACS analysis performed in H1299 cells after 48 hours of transfection with 5 nM miR-145-5p mimic or control mimic.

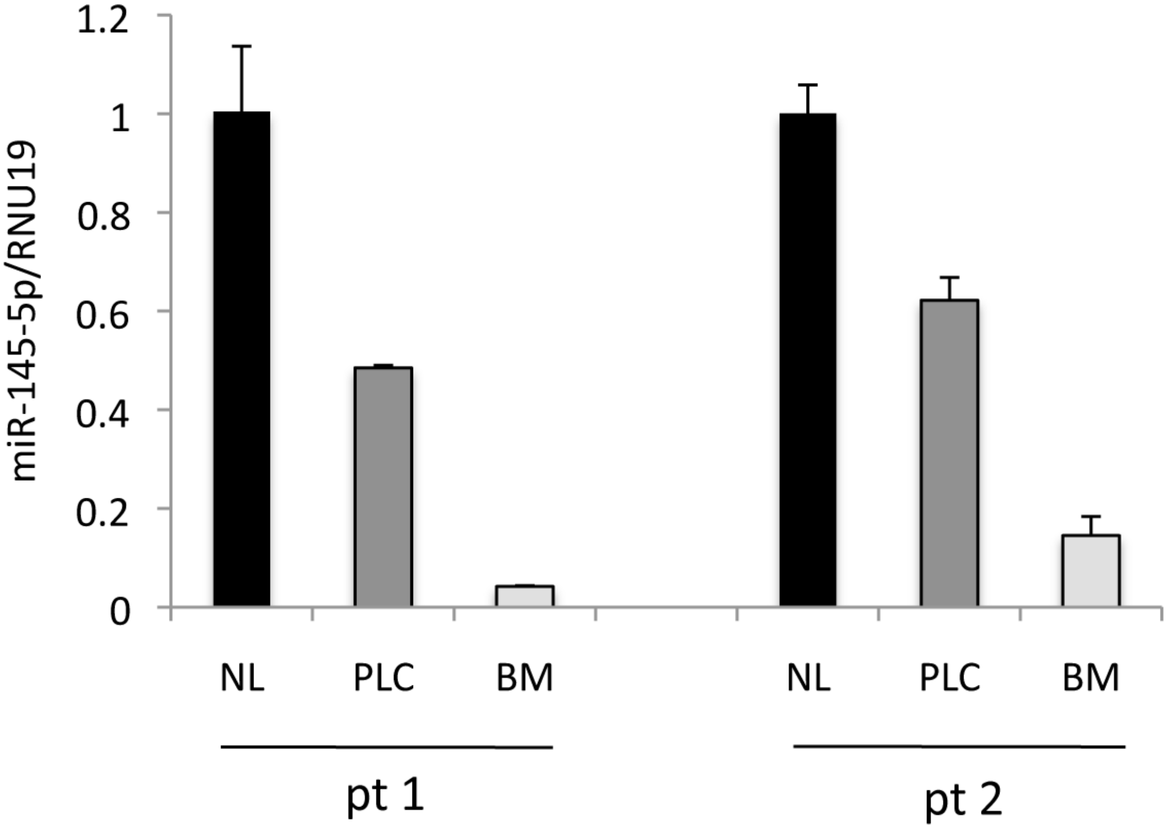

Supplementary figure S8.

qRT-PCR of miR-145-5p expression levels in 2 representative patients of the casuistry used for immunohistochemistry assay (NL= normal lung; PLC= primitive lung cancer; BM= brain metastases; pt= patient).

A

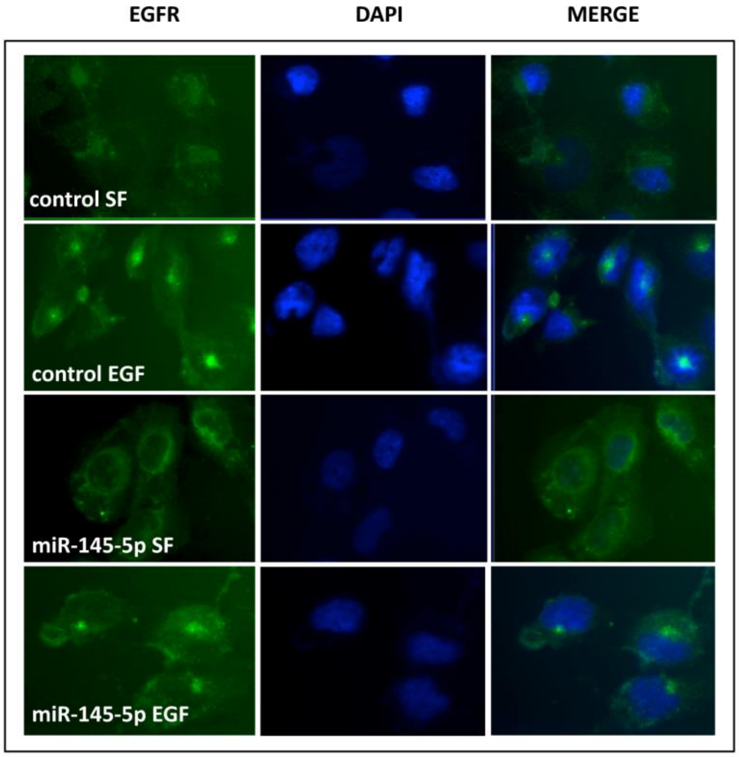

B

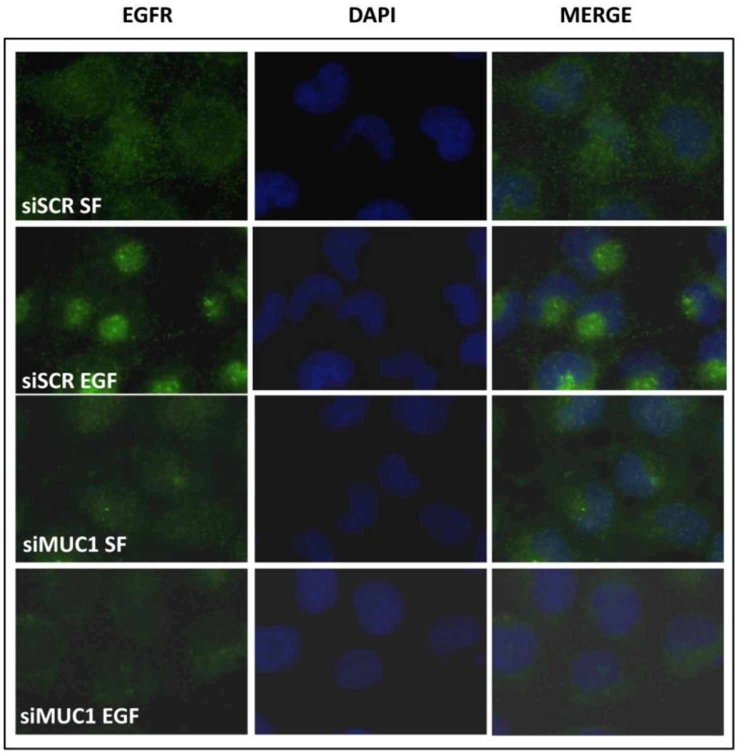

Supplementary figure S9.

A) Immunofluorescence assay to analyze EGFR localization in H1299 cells treated with EGF (20 ng/mL) upon miR-145-5p over-expression. **B)** Immunofluorescence assay and relative quantification to analyze EGFR localization in H1299 cells treated with EGF (20ng/mL) upon MUC-1 RNA interference.

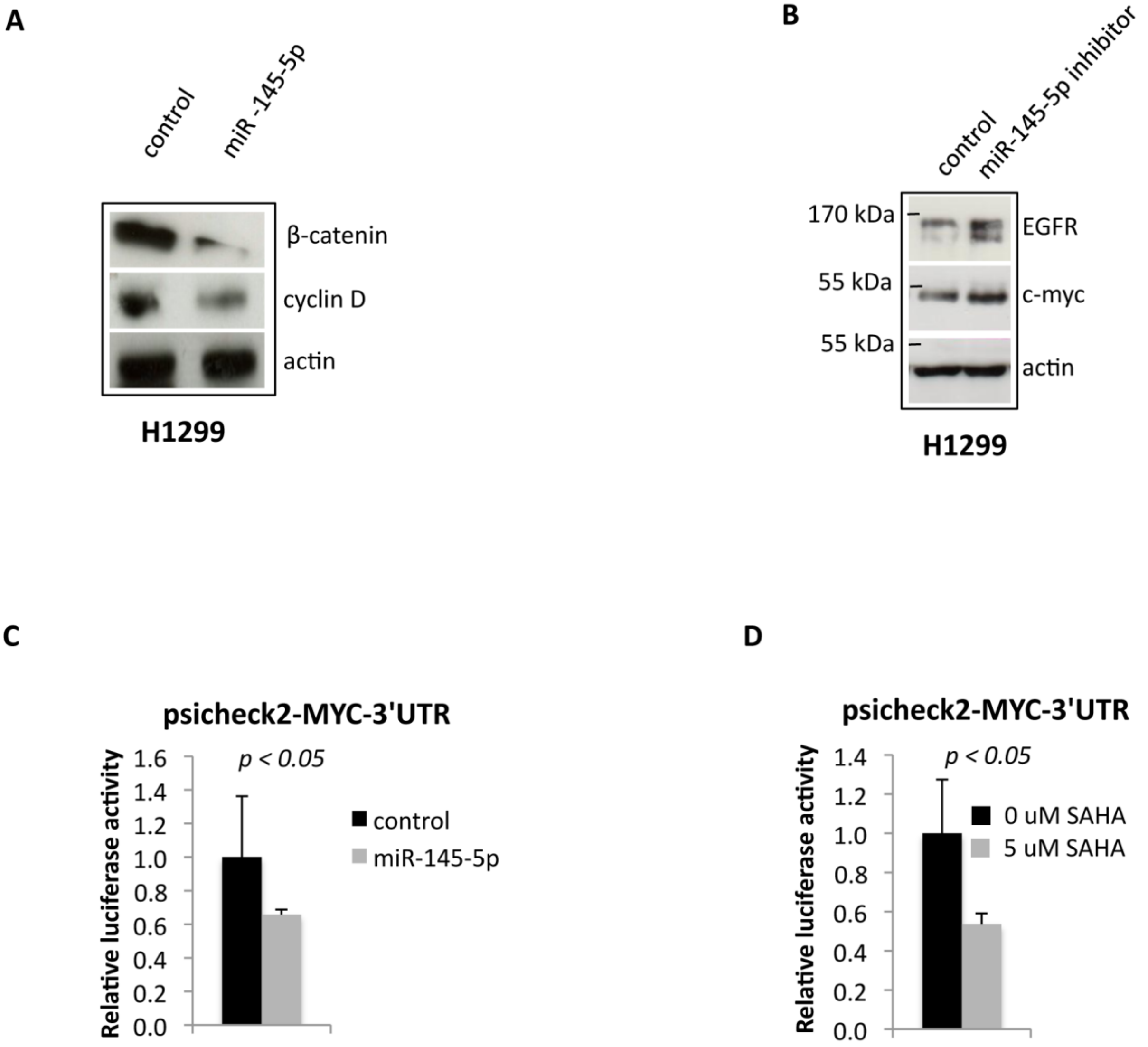

Supplementary figure S10.

A) Western-blot analysis of β-catenin and cyclin-D1 proteins expression levels in H1299 cells upon miR-145-5p over-expression. **B)** Western-blot analysis of EGFR and MYC proteins expression levels in H1299 cells upon miR-145-5p depletion. **C)** Renilla luciferase activity of MYC-3'UTR reporter gene in H1299 transiently transfected with miR-145-5p mimic or control mimic. **D)** Renilla luciferase activity of MYC-3'UTR reporter gene in H1299 treated with 5 uM vorinostat (SAHA).

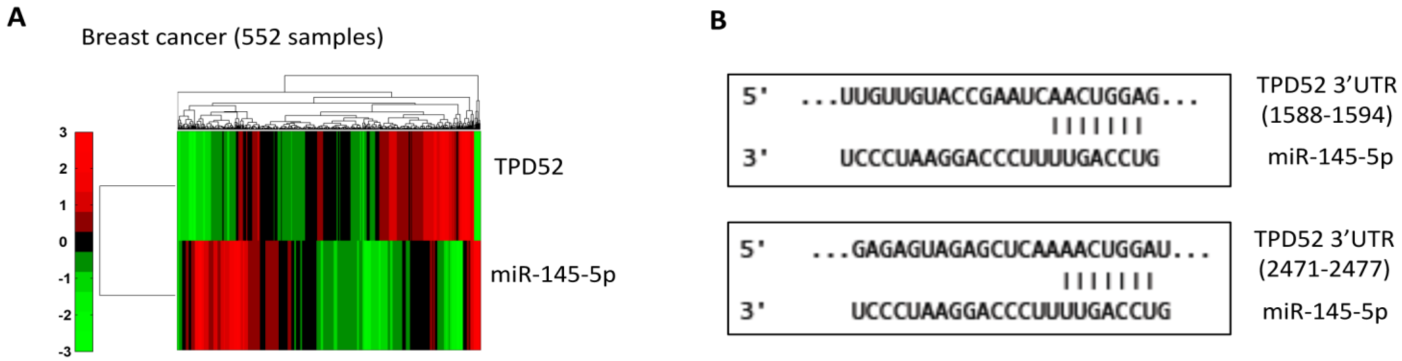

Supplementary figure S11.

A) Heat map of TPD52 and miR-145-5p genes expression in tumoral samples of breast cancer TCGA dataset. **B)** Predicted duplex formation between human TPD52 3' UTR and miR-145-5p.

Supplementary table 1. Primers used for RT-PCR .

Gene		Sequence
Pri-miR-145	FW	5'--- GAGAACTCCAGCTGGTCCTTA---3'
	RV	5'---GGTGGGAAGGAGGCAAAT---3'
GAPDH	FW	5'---GAGTCAACGGATTTGGTCGT---3'
	RV	5'---GACAAGCTTCCCGTTCTCAG---3'

Supplementary Table 2. Primers used for RT-qPCR

Gene		Sequence
MYC	FW	5'---CCTACCCTCTAACGACAGC---3'
	RV	5'---CTCTGACCTTTTGCCAGGAG---3'
EGFR	FW	5'---TCCCTCAGCCACCCATATGT---3'
	RV	5'---AATGACAAGGTAGCGCTGGG---3'
TPD52	FW	5'---GCAAGACGTGACAGCAACAT---3'
	RV	5'---TGGTGATGACTGAGCCAACA---3'
GAPDH	FW	5'---GAGTCAACGGATTTGGTCGT---3'
	RV	5'---GACAAGCTTCCCGTTCTCAG---3'

Supplementary Table 3. Primers used for RT-qPCR in MeDIP experiment

Gene		Sequence
miR 145 CpG island	FW	5'---GAGAACTCCAGCTGGTCCTTA---3'
	RV	5'--- GGTGGGAAGGAGGCAAAT---3'

Supplementary Table 4. Primers used for PCR reaction and sequencing analysis

MiR 145 Region1 a		Sequence
Primer for amplification	FW	5’GGAGATTGGGGAATATATATGAGT
	RV	Biot 5’-TTCTACATCCAACCCCATCTATAACA
Primer for sequencing	S1	Fwd 5’GAGGGTAGTTTTGGG
MiR 145 Region1 b		Sequence
Primer for amplification	FW	5’TGGGGTTGGATGTAGAAGAGAATT
	RV	Biot 5’-TATTTCCAAAAATCCCCATCTTAACAT
Primer for sequencing	S1	Fwd 5’ATTTTAGTTGGTTTTTAGGGATA

Supplemetary Table 5. Primer used for ChIP assay

		Sequence
miR-145-promoter	FW	5’--- CTTGTGATGCTGGGGAAGTT---3’
	RV	5’--- GGGCTCAGAAAGAGAAAGCA---3’
